# Clinical and molecular sub-classification of hepatocellular carcinoma relative to alpha-fetoprotein level in an Asia-Pacific island cohort

**DOI:** 10.20517/2394-5079.2017.46

**Published:** 2018-01-12

**Authors:** Scott T. Nishioka, Miles M. Sato, Linda L. Wong, Maarit Tiirikainen, Sandi A. Kwee

**Affiliations:** 1 The Queen’s Medical Center, Honolulu, HI 96813, USA; 2 Cancer Biology Program and Genomics Shared Resource, University of Hawaii Cancer Center, University of Hawaii, Honolulu, HI 96822, USA; 3 Hamamatsu/Queen's PET Imaging Center, The Queen’s Medical Center, Honolulu, HI 96813, USA

**Keywords:** Hepatocellular carcinoma, alpha-fetoprotein, survival, gene expression, enrichment analysis, molecular signature, Asia-Pacific, Hawaii

## Abstract

**Aim:**

Increased serum alpha-fetoprotein (AFP) levels are associated with specific molecular sub-classes of hepatocellular carcinoma (HCC), supporting AFP as a predictive or therapeutic biomarker for precision treatment of this disease. Considering recent efforts to validate HCC molecular classification systems across different populations, we applied existing signature-based classification templates to Hawaii cohorts and examined whether associations between HCC molecular sub-class, AFP levels, and clinical features found elsewhere can also be found in Hawaii, a region with a unique demographic and risk factor profile for HCC.

**Methods:**

Whole-genome expression profiling was performed on HCC tumors collected from 40 patients following partial hepatectomy. Tumors underwent transcriptome-based categorization into 3 molecular sub-classes (S1, S2, and S3). Patient groups based on molecular sub-class and AFP level were then compared with regards to clinical features and survival. Differences associated with AFP level and other clinical parameters were also examined at the gene signature level by gene set enrichment analysis.

**Results:**

Statistically confident (false discovery rate < 0.05) sub-classifications were made in 98% (39/40) of tumors. Patient sub-groups differed significantly with regards to serum AFP level, with significantly lower levels in the S3 sub-group as compared to S1 (*P* = 0.048) and S2 (*P* = 0.010). Serum AFP > 400 ng/mL predicted significant tumor enrichment for genes corresponding to *MYC* target activation, high cell proliferation, poor clinical prognosis, and the S2 sub-class. AFP > 400 ng/mL and non-S3 tumor classification were found to be significant predictors of overall survival.

**Conclusion:**

Distinct sub-classes of HCC associated with different molecular features and survival outcomes can be detected with statistical confidence in a Pacific Island cohort. Molecular classification signatures and other predictive markers for HCC that are valid for all patient populations are needed to support multi-center efforts to develop targeted therapies for HCC.

## INTRODUCTION

Hepatocellular carcinoma (HCC) remains a leading cause of cancer-related mortality worldwide despite current extensive knowledge about its preventable risk factors^[1,2]^. The highest incidence rates of HCC are in areas with endemic hepatitis B virus (HBV) infection such as China and Sub-Saharan Africa, however HCC incidence has been increasing in Oceania, Europe, and the United States (US) owing to the rising prevalence of other HCC risk factors such as hepatitis C virus (HCV) infection and steatohepatitis^[1,3]^. In the US alone, HCC diagnoses have tripled since 1975, and with a 5-year survival rate as low as 12%, HCC is fast becoming a leading cause of cancer-related mortality in this region^[3]^. Reflecting its predominantly Asian and Pacific Islander demographic, Hawaii has one of the highest incidence rates of liver cancer in the US, with an age-adjusted incidence rate of 11.0 per 100,000 that is considerably higher than the overall US rate of 7.6 per 100,000^[4]^. Given that the distribution of HCC risk factors in Hawaii differs from that of both Asia and the continental US^[5–7]^, studies involving cohorts from Hawaii may provide additional insights into the disease.

HCC is renowned for its genomic and molecular diversity. Recent attempts at HCC molecular sub-classification have produced multiple, sometimes orthogonal, classification systems that associate with various clinical, histological, and molecular features^[8–12]^. The molecular diversity of HCC makes targeted therapy challenging, since it dilutes any individual therapeutic target within the patient population, leading to weaker overall benefit in conventional clinical trials^[13–15]^. Consequently, it is not surprising that among HCC clinical trials to date, all molecularly-specific agents have failed, and only multi-targeting agents such as sorafenib have shown efficacy^[16]^. A robust molecular sub-classification system for HCC could enable clinical trials to enrich study cohorts according to tumoral expression of targeted molecular pathways^[11,15–17]^. In fact, this may be the most important next step in advancing patient-individualized treatment of HCC. It would therefore be prudent to validate HCC sub-classification systems across many different patient populations worldwide.

Serum alpha-fetoprotein (AFP) measurement has been used extensively for HCC screening and diagnosis, despite being associated with a limited diagnostic sensitivity of approximately 66%^[18,19]^. Possibly explaining this limited sensitivity, different molecular sub-classes of HCC have been associated with different degrees of AFP production^[10–12]^. Clinically, differences in AFP production have also been associated with gross and histopathologic tumor differences, including differences in tumor size, multinodular appearance, and vascular invasion^[20]^. AFP may also be directly involved in tumor pathogenesis through its involvement in several mitogen and anti-apoptotic pathways, as well as potentially by exerting paracrine effects on immune and other non-tumor cells^[21,22]^. Given these associations, AFP could have significant value beyond that of a diagnostic marker. While several molecular classification systems for HCC have been associated with differences in AFP levels across their respective sub-classes^[10,11]^, these associations along with the classification systems themselves are in need of further validation across many different population cohorts. Since Hawaii has a unique and diverse patient population, we assessed the feasibility of applying HCC molecular classification systems derived from other patient populations to those in Hawaii and examined the relationship of AFP and other clinical parameters to the transcriptomic features of HCC.

## METHODS

### Tissue samples

Forty patients diagnosed with BCLC stage A HCC who were referred to a single medical center for primary treatment of HCC by partial hepatectomy were prospectively recruited to participate in an institutional review-board approved clinical research study with written informed consent. All patients were deemed clinical candidates for hepatic resection by an attending surgeon, and a separate informed consent process for surgery was completed before study recruitment.

### Whole transcriptome analysis

At the time of surgery, tumor and adjacent non-tumor samples were taken from the resection specimen and conserved in separate containers with RNA Later medium (Thermo Fisher, Waltham, MA). RNAs were subsequently extracted from homogenized frozen liver tissue lysates in RLT Plus buffer with the All Prep DNA/RNA Mini kit (Qiagen, Valencia, CA). The isolated RNAs were then stored at −80 °C until analysis.

The analytical quality of the total RNAs was assayed using a Bioanalyzer with RNA 6000 Nano chips (Agilent, Santa Clara, CA) prior to use for this study. Isolated RNAs were then processed following the WG-DASL assay protocol (Illumina Inc., Sunnyvale, California). Resulting PCR products were hybridized onto the Illumina HumanHT-12 v4 Expression Bead Chips covering over 24,000 transcripts with genome-wide coverage of well-characterized genes, gene candidates, and splice variants. Arrays were scanned using the iScanTM instrument and expression levels were quantified using Genome Studio software (Illumina Inc., Sunnyvale, CA). The resulting expression data matrix contained 40 columns representing individual tumor samples and 20,818 rows corresponding to gene expression data.

This gene expression dataset was pre-processed by generalized log2 transformation with background subtraction, quantile normalization, and row centering. Each sample was annotated with corresponding clinical data such as age, gender, FIB-4 score, AFP level, and HCC risk factor data, as obtained from clinical records. All tumor samples, gene expression data, and clinical parameters were de-identified and assigned a serial number to maintain patient confidentiality.

### Tumor classification based on gene expression signature

Tumor molecular classification was based on the Hoshida system, using sub-classification signatures previously subjected to meta-analysis in 6 different patient cohorts collected from 3 continents (Asia, Europe, and North America)^[10]^. Based on this classification system, samples were categorized by nearest template into 3 distinct HCC sub-classes (labeled S1, S2, and S3)^[23]^. A false discovery rate (FDR) < 0.05 was used as the statistical criterion for confident sub-class label assignments.

### Clinical classification

The histologic diagnosis of HCC was established for each patient by clinical pathology. These diagnoses were further confirmed in all tumor samples by a single board-certified hepatobiliary pathologist. Tumor samples were then sub-categorized based on several clinical parameters to be later used as classes for gene set enrichment analysis (GSEA). These categorizations were based on the distribution of each clinical parameter for all tumor samples. The clinical parameters to be used as class phenotype labels were selected a priori. They were age, gender, FIB-4 score, AFP level, and presence of HBV infection. Except for gender and HBV infection, which are binary, parameters were dichotomized for GSEA based on analysis of dispersion. AFP levels displayed a bimodal distribution so that a cut-off point between “high” and “low” AFP values could be made at the histogram trough corresponding to 400 ng/mL. Coincidentally, an AFP cut-off point of 400 ng/mL is frequently used clinically as a highly specific cut-off for confirming HCC diagnosis^[24]^, and also frequently serves as a cut-off point for determining eligibility in clinical trials involving agents with potential selectivity for AFP-producing tumors (e.g. NCT02435433). In contrast, the distribution of FIB-4 scores was highly skewed and did not fit a normal or bimodal distribution to provide a logical location for the cut-off point. A FIB-4 cut-off was therefore prospectively chosen based on review of previous literature regarding FIB-4 scores and their prognostic value. A study conducted by Chan *et al*.^[16]^, which aimed to determine an optimal cut-off point for diagnosing and prognosticating advanced liver fibrosis after curative liver resection in HCC patients found that a FIB-4 index of 2.87 optimized both sensitivity and specificity. As a result, samples were dichotomized based on a FIB-4 score of 2.87.

### GSEA

GSEA was used to test the hypothesis that gene expression profiles corresponding to a priori defined gene sets differ between samples belonging to 2 distinct phenotype classes^[25]^. Using a Java-based implementation of the GSEA algorithm (GSEA v3.0, Broad Institute, Boston, MA), the enrichment of gene sets of interest within tumors corresponding to a given clinical phenotype were sought. To perform significance testing against a null-hypothesis, permutation testing was performed to compute enrichment scores for 1000 random phenotype assignments. A FDR of less than 0.25 was used to indicate significant enrichment and prompt further inquiry about tumor biology using biomedical literature referenced in the GSEA output.

Current versions (v6.0) of curated collections of gene sets were downloaded from an online database MSigDB (MSigDB, Broad Institute, Boston, MA) from within the GSEA Java application. The Hallmarks collection (comprised of 50 gene sets composed of coherently expressed genes reflecting well-defined biological states or processes) and the chemical and genetic perturbations (CGP) collection (comprised of 2675 gene sets reflecting gene signatures derived from published biomedical literature) were used for this study. The CGP collection includes gene signatures reflecting genetic and chemical perturbations from a broad variety of diseases. To estimate the number of HCC-related gene sets in the CGP collection, a query for “hepatocellular carcinoma” was performed using the search mechanism of the mSigDB online portal (http://software.broadinstitute.org/gsea/msigdb/index.jsp). This revealed 107 gene sets within the CGP collection related to HCC that were supported by literature from Medline-indexed journals. These gene sets included multiple published gene signatures for HCC molecular classification^[8,10,11,26]^ and prognostication^[12,27]^.

### Statistical methods

Differences involving normally distributed variables were assessed by *t*-test or analysis of variance. *Post hoc* multiple pair wise comparisons were performed by the Steel-Dwass test. Comparisons among categorical or dichotomized variables were assessed using Fisher’s exact test. Kaplan-Meier analysis was used to compare overall survival rates post-surgical resection in patients stratified by AFP > 400 ng/mL and AFP ≤ 400 ng/mL, and by combined S1 and S2 tumor subclasses *vs.* S3 subclass. Differences in survival curves were assessed using the Log-Rank tests. Cox proportional hazard ratios were also computed for the effects of AFP level differences and tumor class differences on overall survival post-surgical resection. Adjustments to proportional hazards regression models were made only if multiple significant univariate predictors of overall survival were identified. All statistical analyses were carried out using SAS version 9.3 (SAS Institute, Cary, NC).

## RESULTS

Patient clinical characteristics and demographics (*n* = 40) are summarized in Table 1. There were no significant differences in various clinical parameters including age, gender, HBV infection, HCV infection, significant alcohol use, Edmondson-Steiner grade, or proportion of high FIB4 scores between the AFP > 400 ng/mL and AFP ≤ 400 ng/mL groups of patients [Table 2].

### Tumor classification

**Figure 1:**
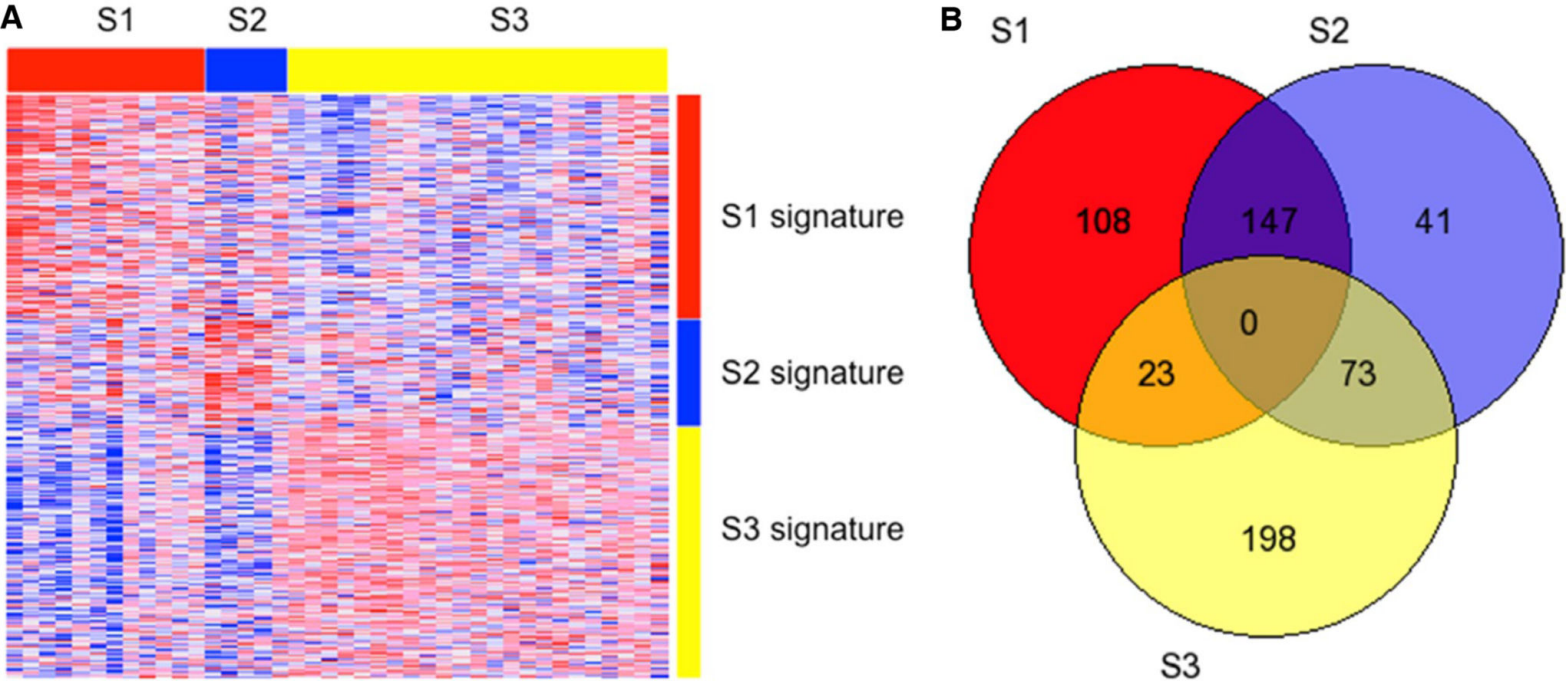
(A) Clustered heat map showing expression pattern of the HCC subclassification signature among 39 patients. Columns represent tumor samples clustered into S1 (red), S2 (blue), and S3 (yellow) HCC sub-classes. Rows represent genes comprising the S1 (red), S2 (blue), and S3 (yellow) classification signatures. Confident prediction (FDR < 0.05) occurred in 98% (39/40) of cases; (B) Venn diagram showing the number of HCC sub-classification genes that are common and differentially expressed among the S1, S2, and S3 sub-classes. HCC: hepatocellular carcinoma; FDR: false discovery rate

The number of tumors mapped into tumor sub-class S1, S2, and S3, were 12, 4, and 23 respectively. Only one tumor could not be classified based on a FDR < 0.05. The remaining sub-class assignments were also statistically significant based on Bonferroni-corrected *P*-values < 0.05. A heat map depicting classification signature expression patterns and a Venn diagram summarizing the number of differentially expressed signature genes between sub-classes are shown in Figure 1. Corresponding serum AFP levels differed significantly across tumor sub-classes (Wilcoxon *P* = 0.002). Post hoc pair wise testing adjusted for multiple comparisons revealed significant differences in AFP levels between sub-classes S3 and S1 (72 *vs.* 2332 ng/mL, *P* = 0.048) and between S3 and S2 (72 *vs.* 4277 ng/mL, *P* = 0.010). Functional annotation of the sub-classification results by Gene Ontology Biological Processes is shown in Supplementary Table 1.

### GSEA results

**Figure 2:**
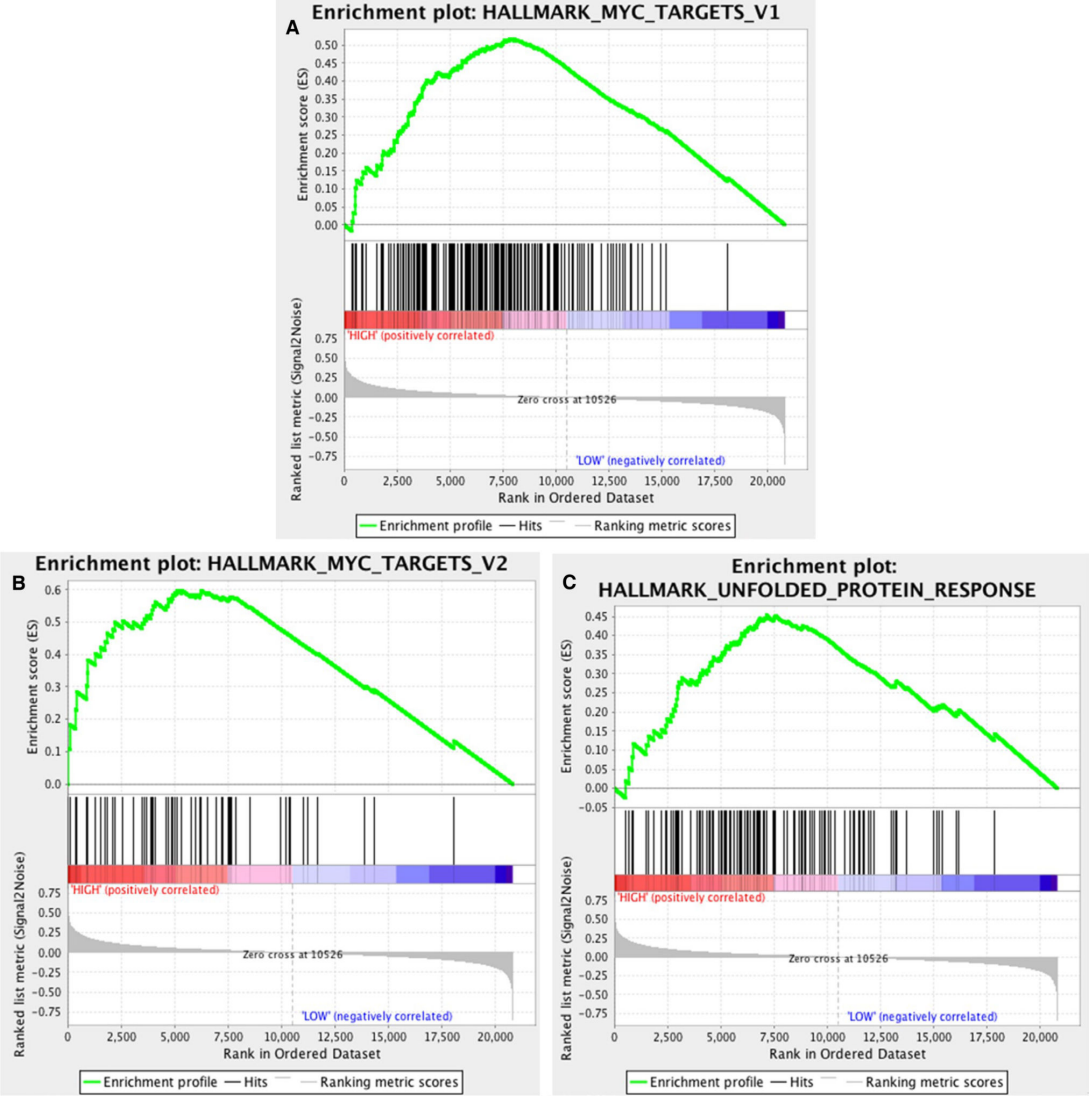
Profile of the running enrichment score and position of gene set members on the rank ordered list for (A) MYC_TARGETS_V1; (B) MYC_TARGETS_V2; and (C) UNFOLDED_PROTEIN_RESPONSE from the HALLMARKS gene set collection. The two MYC_TARGETS gene sets (comprised of 200 and 58 genes, respectively) share only 18 genes in common

In comparing HCC tumors associated with serum AFP > 400 ng/mL (high AFP class) with those associated with lower AFP levels, multiple gene sets from the Hallmarks and CGP collections were significant based on FDR < 0.25. From the Hallmarks collection, 7/50 gene sets were identified as significantly enriching the elevated AFP class of tumors. These gene sets are summarized in Supplementary Table 2. Two of the top scoring gene sets from this collection, MYC_TARGETS_V1 and MYC_TARGETS_V2 (with FDR 0.057 and 0.077, respectively), are comprised of genes known to be upregulated in response to MYC oncogene activation. Their enrichment score plots are shown in Figure 2A and B, respectively. These gene sets are relatively independent of each other since they hold only 18 genes in common and are comprised of 200 and 58 genes respectively. Thus, their mutual significance compounds support that the tumors in the high AFP class are enriched for genes controlled by MYC, a finding that is also consistent with previous literature implicating MYC oncogene activation in the pathogenesis of HCC tumors associated with high serum AFP levels^[10]^.

Another top ranked gene set from the Hallmarks collection was UNFOLDED_PROTEIN_RESPONSE (FDR 0.069 and family-wise error rate *P* = 0.044). The enrichment score plot for this gene set is shown in Figure 2C. This gene set is comprised of genes associated with unfolded protein response (UPR). There is recent evidence to suggest that AFP production is proteostatically regulated in part by UPR, although all exact mechanisms have not yet been clarified^[28]^. In one study, exposure of HCC cells to sorafenib led to changes in UPR that affected AFP production independent of an effect on cell viability, a finding that suggests that AFP could potentially serve as a biomarker of tumor proteostatic response^[29]^. The remaining significant gene sets from the Hallmarks collection (E2F_TARGETS, G2M_CHECKPOINT, DNA_REPAIR, and MITOTIC_SPINDLE) were all found to relate to cell proliferation, as the E2F transcription factory family is known to integrate cell cycle progression with DNA repair, replication, and G2/M checkpoints^[30]^. A heat map based on the list of ranked genes from GSEA using the Hallmarks gene set collection is shown in Supplementary Figure 1.

GSEA using the CGP collection identified 351 gene sets as being significantly enriched in the high AFP class of tumors. These gene sets and their corresponding significance and enrichment scores are summarized in Supplementary Table 3. Although this collection (comprised of 2675 signatures derived from a broad variety of diseases and conditions) included relatively few HCC-related gene sets, a disproportionate number of them were found to be significant [Table 3].

Several of the gene signatures found to be significant have previously been associated with high AFP levels, including HOSHIDA_LIVER_CANCER_SUBCLASS_S2^[10]^, CHIANG_LIVER_CANCER_SUBCLASS_PROLIFERATION_UP^[8]^, and YAMASHITA_LIVER_CANCER_WITH_EPCAM_UP^[12]^. In addition to these HCC-specific gene sets, 14 gene sets from the CGP collection representing MYC target genes were also found to be significantly enriched in the high AFP class, which falls in agreement with the enrichment analysis results obtained using the Hallmarks collection. A heat map of the top ranked genes from this analysis is shown in Supplementary Figure 2. No significant differences in gene set enrichment were found between phenotype classes determined by age, gender, FIB4 or HBV-status using either the Hallmarks or CGP collections.

### Survival analysis

**Figure 3:**
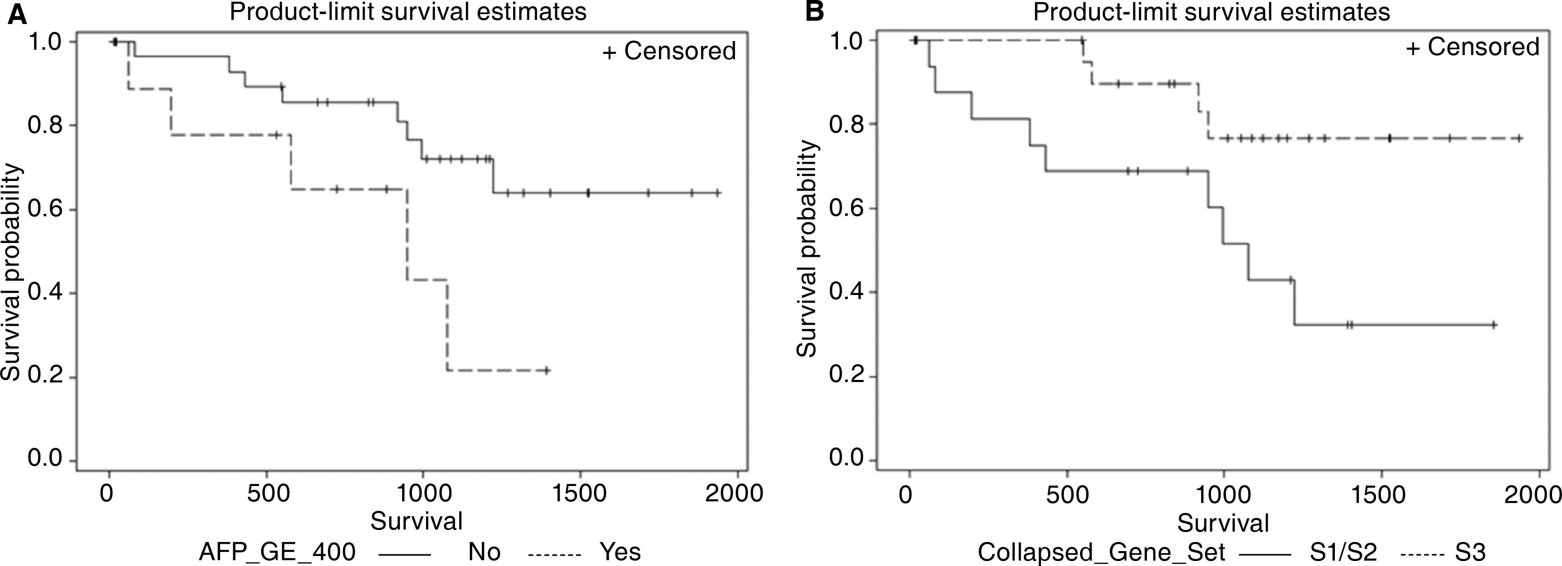
Kaplan-Meier survival rate curves showing significant survival differences among (A) patients with AFP > 400 ng/mL (AFP_GE_400) *vs.* ≤ 400 ng/mL (Log-Rank *P* = 0.040), and among (B) patients with tumor subclass S1 & S2 *vs.* tumor subclass S3 (Log-Rank *P* = 0.024)

A Kaplan-Meier plot comparing the survival of patients with serum AFP level > 400 ng/mL versus patients with lower serum AFP levels is shown in Figure 3A. Serum AFP level > 400 ng/mL was significantly associated with poorer overall survival (Log-Rank *P* = 0.040) and a hazard ratio for mortality of 3.1 (95%CI 1.0–9.7, *P* = 0.050).

For survival analysis based on the molecular classification of tumors, patients whose tumors were assigned to sub-classes S1 and S2 were grouped together because of the similarity in gene expression between sub-classes S1 and S2 relative to S3 [Figure 1] and the significant associations of sub-classes S1 and S2 with higher AFP levels (mean 2819 ng/mL) as compared to S3 (mean 72 ng/mL). A Kaplan-Meier plot comparing the survival rates of patients with non-S3 tumors *vs.* patients with S3 tumors is shown in Figure 3B. Non-S3 tumors were significantly associated with poorer overall survival (Log-Rank *P* = 0.024) and a mortality hazard ratio of 3.6 (95% confidence interval 1.1–11.6, *P* = 0.035). Age, gender, FIB4 > 2.87, and HBV infection were not found to be significant predictors of overall survival following liver resection.

## DISCUSSION

In this study, tumoral differences were examined at the gene signature level between HCC sub-groups categorized on the basis of AFP and other clinical parameters. Using GSEA, we found no significant differences in gene set enrichment between tumors categorized by patient age, gender, clinical severity of liver fibrosis, and HBV infection. However, we did find significant differences between tumors categorized by patient serum AFP level. These differences proved to be biologically coherent across analyses involving two distinct gene set collections from the mSigDB molecular signature repository. Specifically, using the mSigDB Hallmarks collection of gene sets, we found serum AFP levels > 400 ng/mL to be associated with gene set enrichments corresponding to MYC oncogene activation, enhanced DNA replication/repair, and cell cycle progression, all of which are defining properties of highly proliferating tumors. In addition, we found tumors from patients with high serum AFP levels to be significantly enriched for genes associated with proteostasis, a potential mechanism for the release of AFP by tumor cells^[22]^.

Using the larger *CGP* gene set collection comprised of 2675 gene signatures, we found that tumors associated with high serum AFP levels were also significantly enriched for genes belonging to several existing molecular classification signatures for HCC. Three of these signatures have already been associated with high AFP levels by previous studies. The first signature corresponds to the S2 tumor sub-class defined by Hoshida *et al*.^[10]^. In addition to being associated with high serum AFP levels, this sub-class of HCC tumors is characterized by MYC oncogene activation and enhanced cellular proliferation. Thus, these results are concordant with the results obtained by GSEA using the Hallmarks gene set collection. Another HCC sub-classification signature found significantly enriched in the high AFP class of tumors corresponds to a “proliferation” sub-class of HCC described by Chiang *et al*.^[8]^. In addition to being associated with high serum AFP levels, this sub-class is associated with chromosomal instability and overexpression of proliferation-related genes. The third HCC classification signature that was significant in our analysis corresponds to an EpCAM signature defined by Yamashita *et al*.^[12]^. In their study, this signature, when combined with AFP expression, identifies four patient sub-groups, each with their own unique sub-signature and survival pattern. Notably, the AFP-positive sub-groups were associated with higher TNM stage and worse clinical prognosis^[12]^. Altogether, these distinct signatures (from three different molecular classification systems) were consistent in ascribing aggressive biological traits to the high AFP class of tumors in our study.

Supporting the premise that aggressive tumor biology leads to worse clinical outcomes, we also found the LEE_LIVER_CANCER_SURVIVAL_DN gene signature (comprised of genes highly expressed in HCC associated with poor survival^[27]^), to be significantly enriched in the high AFP class of tumors from our study. Confirming this prognostic result, we found overall survival rates of patients with AFP levels > 400 ng/mL to be significantly lower than those with AFP level ≤ 400 ng/mL. Furthermore, survival analysis based on molecular tumor classification revealed significantly lower survival rates in patients with tumor sub-classes associated with high AFP levels.

Of course, clinical associations between high AFP levels and poor prognosis in HCC are not unique to our study. Elevations in AFP have been widely shown to predict poorer prognosis, especially when used in combination with other clinical markers^[31]^. We also previously found an association between increased AFP levels (> 400 ng/mL) and HCC recurrence following liver transplantation^[24]^. Our study contributes to this existing knowledge by showing the potential of functional genomics to link clinical measurements of serum AFP to the molecular mechanisms of AFP production, other tumor biological traits, and molecular tumor classification to provide clues on therapeutic target enrichment and treatment outcome in an understudied patient population.

Because many contemporary clinical guidelines do not require a histopathological diagnosis before treatment of HCC, tumor tissue is often not available for molecular profiling in the clinical setting. This inadvertently poses a barrier to routine molecular classification of HCC based on tumor tissue. While liquid biopsy techniques are being developed to profile HCC circulating tumor cells and associated cell-free DNA, serum AFP remains the most readily available hematogenous biomarker for HCC. Because of associations between AFP expression and the expression of other potential cellular targets^[11,12,32]^, serum AFP may have value as a surrogate predictive biomarker for molecularly-targeted therapy. In pre-clinical studies, differences in AFP expression have already been correlated with differences in therapeutic response. For example, differences in response to the Src/Abl kinase inhibitor, dasatinib have been observed between AFP-positive and AFP-negative HCC cell lines^[33]^. AFP expression status has also shown high correlation with specific HCC molecular subtypes in different cell lines^[34]^. Thus, AFP expression status is an important variable for interpreting the results of both pre-clinical studies and clinical trials of HCC.

Several limitations of the present study should be recognized. First, the tumor samples analyzed in this study were collected from patients recruited from a single medical center in the state of Hawaii. This raises the possibility of selection bias. However, the likelihood of such bias is reduced given the prospective nature of this study and the fact that our center is responsible for treating most of the HCC patients in Hawaii. Because Hawaii is a small territory, the number of patients presenting annually with HCC is also relatively small despite the high incidence, and thus the statistical power of this study is limited. However, unlike unsupervised methods, GSEA has been found to be fairly robust with sample size in the range of the present study^[35]^. Another potential limitation relates to the fact that gene expression analysis was performed by sampling only a small peripheral portion of the tumor. Because of this, our results cannot account for the possibility of intra-tumoral heterogeneity. Notwithstanding this methodologic limitation, the results of this study did produce a biologically-coherent depiction of HCC tumors associated with high serum AFP levels.

Our study provides additional data supporting the clinical relevance of gene signatures for HCC derived from many different cohorts, including those from Asia, Europe, and North America. Because there are studies suggesting that ancestry and genetics may influence HCC genomes^[36]^, it is prudent to validate predictive gene signatures for HCC in a broad spectrum of patients before accepting them into mainstream application. While some gene signatures for HCC have already been subject to further validation^[10]^, none have been thoroughly validated to a global extent. Our study, conducted in a racially and ethnically diverse HCC cohort, provides further evidence to support the generalizability of gene signatures for clinical molecular classification of HCC. Specifically, we confirmed that several externally derived molecular subclasses of HCC associated with distinct molecular features and survival outcomes could be detected with statistical confidence in a cohort of patients from Hawaii. The generalization of these signatures will support their use in multi-center efforts aimed at developing targeted therapies for HCC.

In conclusion, herein we provide supporting evidence that a molecular classification system for HCC developed using cohorts from North America, Europe, and Asia is applicable to patients in Hawaii. Similar to other cohorts, the findings in the present study also indicate that elevated AFP is significantly associated with more aggressive tumor characteristics and poor clinical outcome, as well as gene expression related to cell cycle progression, DNA damage response, and MYC oncogene pathways. Confirming the ability to apply the same molecular classification system to tumors from different populations is a crucial step to broadening the use of genomic enrichment strategies in global multi-center clinical trials. Establishing that similar distributions of tumor sub-classes exist in different populations will also increase confidence that molecularly-targeted therapies found to be beneficial in one cohort can be similarly effective in cohorts from other populations.

## Supplementary Material

Supplemental Results

## Figures and Tables

**Table 1:** Clinical characteristics and demographics of the patient cohort

Characteristics	Data
No. of patients	40
Mean age, years	64.0
Gender, male/female	30/10
HBV-infected, *n* (%)	5 (12.5%)
HCV-infected, *n* (%)	9 (22.5%)
Alcohol abuse, *n* (%)	3 (7.5%)
Combination HBV/alcohol, *n* (%)	5 (12.5%)
Combination HCV/alcohol, *n* (%)	8 (20.0%)
Racial cateogry	
Asian	21
Native Hawaiian/Pacific Islander	9
White	8
Black/African American	2

HBV: hepatitis B virus; HCV: hepatitis C virus

**Table 2:** Comparison of clinical characteristics between patients with serum AFP > 400 ng/mL and lower AFP values

Characteristics	AFP (ng/mL)				*P*-value

		> 400		≤ 400	
No. of patients		9		31	
Mean age, years		67.1		62.9	0.314
Gender	Male		6 (66.7%)	24 (77.4%)	0.665
	Female		3 (33.3%)	7 (22.6%)	
FIB4 score	≥ 2.87		3 (33.3%)	15 (48.4%)	0.476
	< 2.87		6 (66.7%)	16 (51.6%)	
Edmondson	ES 1		0 (0.0%)	3 (9.7%)	0.351
- Steiner Grade	ES 2		3 (33.3%)	18 (58.1%)	
	ES 3		5 (55.6%)	8 (25.8%)	
	ES 4		1 (11.1%)	2 (6.5%)	
Risk factors	HBV		0 (0.0%)	5 (16.1%)	0.522
	HCV		3 (33.3%)	6 (19.4%)	
	Alcohol		0 (0.0%)	3 (9.7%)	
	HBV/alcohol		1 (11.1%)	4 (12.9%)	
	HCV/alcohol		1 (11.1%)	7 (22.6%)	
	None		4 (44.4%)	6 (19.4%)	

AFP: alpha-fetoprotein; HBV: hepatitis B virus; HCV: hepatitis C virus

**Table 3:** HCC-related gene sets from the chemical and genetic perturbations collection enriched in the high AFP tumor class

Name	FDR
BOYAULT_LIVER_CANCER_SUBCLASS_G3_UP	0.086
HOSHIDA_LIVER_CANCER_SUBCLASS_S2	0.088
BOYAULT_LIVER_CANCER_SUBCLASS_G123_UP	0.099
CHIANG_LIVER_CANCER_SUBCLASS_PROLIFERATION_UP	0.101
YAMASHITA_LIVER_CANCER_WITH_EPCAM_UP	0.104
SAKAI_CHRONIC_HEPATITIS_VS_LIVER_CANCER_UP	0.118
LEE_LIVER_CANCER_SURVIVAL_DN	0.147
CHIANG_LIVER_CANCER_SUBCLASS_UNANNOTATED_DN	0.186
BOYAULT_LIVER_CANCER_SUBCLASS_G23_UP	0.228
BOYAULT_LIVER_CANCER_SUBCLASS_G12_UP	0.252

HCC: hepatocellular carcinoma; FDR: false discovery rate
