# Supplementary material for: Clinical and molecular sub-classification of hepatocellular carcinoma relative to alpha-fetoprotein level in an Asia-Pacific island cohort
Source: Hepatoma Res. Author manuscript; Available in PMC 2018 Jan 26. (PMC5786161; doi:10.20517/2394-5079.2017.46)

Supplementary Materials

Supplementary Table 1: Functional annotation of the differentially expressed classification genes in S1 vs. non-S1, S2 vs. non-S2, and S3 vs. non-S3. Annotations are based on Gene Ontology (GO) Biological Process terms.

| Top upregulated functions based on differential expression in S1 versus non-S1 tumors | Count | PValue | Fold Enrichment | FDR |
| --- | --- | --- | --- | --- |
| GO:0007165~signal transduction | 50 | 2.63E-10 | 2.698394333 | 4.49E-07 |
| GO:0030198~extracellular matrix organization | 19 | 2.42E-09 | 6.073865367 | 4.12E-06 |
| GO:0030574~collagen catabolic process | 12 | 4.72E-09 | 11.74813433 | 8.05E-06 |
| GO:0030199~collagen fibril organization | 9 | 1.48E-07 | 14.45924225 | 2.52E-04 |
| GO:0006935~chemotaxis | 12 | 3.95E-06 | 6.162955713 | 0.006737382 |
| GO:0006955~immune response | 21 | 1.40E-05 | 3.125394406 | 0.023884388 |
| GO:0019886~antigen processing and presentation of exogenous peptide antigen via MHC class II | 10 | 1.56E-05 | 6.810512654 | 0.026613043 |
| GO:0050900~leukocyte migration | 11 | 2.52E-05 | 5.64937607 | 0.042981754 |
| GO:0044319~wound healing, spreading of cells | 5 | 2.80E-05 | 26.10696517 | 0.047727495 |
| GO:0006915~apoptotic process | 24 | 3.96E-05 | 2.652136145 | 0.067470714 |
| GO:0001817~regulation of cytokine production | 5 | 3.99E-05 | 24.09873708 | 0.068072539 |
| GO:0071407~cellular response to organic cyclic compound | 8 | 4.00E-05 | 8.495825955 | 0.068232494 |
| GO:0038063~collagen-activated tyrosine kinase receptor signaling pathway | 4 | 7.67E-05 | 41.77114428 | 0.130840782 |
| GO:0031100~organ regeneration | 7 | 9.49E-05 | 9.331851381 | 0.161850239 |
| GO:0006954~inflammatory response | 18 | 1.25E-04 | 2.975780727 | 0.212296707 |
| GO:0050852~T cell receptor signaling pathway | 11 | 1.30E-04 | 4.656918112 | 0.22120312 |
| GO:0007155~cell adhesion | 20 | 1.45E-04 | 2.730140149 | 0.247475496 |
| GO:0001934~positive regulation of protein phosphorylation | 10 | 1.96E-04 | 4.933599718 | 0.334635146 |
| GO:0007568~aging | 11 | 3.14E-04 | 4.177114428 | 0.535065098 |
| GO:0001501~skeletal system development | 10 | 3.46E-04 | 4.57348295 | 0.588855402 |
| GO:0090023~positive regulation of neutrophil chemotaxis | 5 | 3.65E-04 | 14.24016282 | 0.620536602 |
| GO:0043066~negative regulation of apoptotic process | 19 | 3.70E-04 | 2.616434312 | 0.629515617 |
| GO:0042102~positive regulation of T cell proliferation | 7 | 3.73E-04 | 7.309950249 | 0.6337275 |
| GO:0030168~platelet activation | 9 | 5.02E-04 | 4.903569111 | 0.853256233 |
| GO:0042476~odontogenesis | 5 | 8.22E-04 | 11.60309563 | 1.393550657 |
| GO:0035987~endodermal cell differentiation | 5 | 8.22E-04 | 11.60309563 | 1.393550657 |
| GO:0045860~positive regulation of protein kinase activity | 6 | 8.70E-04 | 7.998729755 | 1.474423546 |
| GO:0048010~vascular endothelial growth factor receptor signaling pathway | 7 | 9.92E-04 | 6.091625207 | 1.679372145 |
| GO:0007229~integrin-mediated signaling pathway | 8 | 0.001018849 | 5.063169003 | 1.724157042 |
| GO:0043123~positive regulation of I-kappaB kinase/NF-kappaB signaling | 10 | 0.001109028 | 3.891721517 | 1.875407228 |
| GO:0042554~superoxide anion generation | 4 | 0.001270723 | 17.90191898 | 2.146057397 |
| GO:0031295~T cell costimulation | 7 | 0.001507087 | 5.623038653 | 2.540428077 |
| GO:0007166~cell surface receptor signaling pathway | 13 | 0.001508464 | 2.972763918 | 2.542720326 |
| GO:0031663~lipopolysaccharide-mediated signaling pathway | 5 | 0.001582962 | 9.79011194 | 2.66670673 |
| GO:0001503~ossification | 7 | 0.001717383 | 5.482462687 | 2.890045071 |
| GO:0001666~response to hypoxia | 10 | 0.001755459 | 3.64283235 | 2.953219528 |
| GO:0070374~positive regulation of ERK1 and ERK2 cascade | 10 | 0.001975823 | 3.580383795 | 3.318084739 |
| GO:0033572~transferrin transport | 5 | 0.002220543 | 8.950959488 | 3.721762454 |
| GO:0002504~antigen processing and presentation of peptide or polysaccharide antigen via MHC class II | 4 | 0.002291624 | 14.7427568 | 3.838717843 |
| GO:0042531~positive regulation of tyrosine phosphorylation of STAT protein | 3 | 0.002440338 | 37.59402985 | 4.082973256 |
| GO:0006928~movement of cell or subcellular component | 7 | 0.002483154 | 5.09996529 | 4.15318774 |
| GO:0048013~ephrin receptor signaling pathway | 7 | 0.002483154 | 5.09996529 | 4.15318774 |
| GO:0034142~toll-like receptor 4 signaling pathway | 4 | 0.002717864 | 13.92371476 | 4.537236849 |
| GO:0030335~positive regulation of cell migration | 10 | 0.002772964 | 3.405256327 | 4.627184178 |

| Top upregulated functions based on differential expression in S2 versus non-S2 tumors | Count | PValue | Fold Enrichment | FDR |
| --- | --- | --- | --- | --- |
| GO:0019083~viral transcription | 10 | 4.37E-05 | 5.997142857 | 0.074534848 |
| GO:0000184~nuclear-transcribed mRNA catabolic process, nonsense-mediated decay | 10 | 7.03E-05 | 5.644369748 | 0.119916941 |
| GO:0007165~signal transduction | 35 | 1.08E-04 | 2.024875108 | 0.18480215 |
| GO:0006413~translational initiation | 10 | 2.07E-04 | 4.902773723 | 0.352320222 |
| GO:0006935~chemotaxis | 9 | 4.70E-04 | 4.955016393 | 0.799275817 |
| GO:0006614~SRP-dependent cotranslational protein targeting to membrane | 8 | 4.96E-04 | 5.716425532 | 0.842413348 |
| GO:0000165~MAPK cascade | 13 | 5.60E-04 | 3.332763359 | 0.950898159 |
| GO:0044319~wound healing, spreading of cells | 4 | 6.42E-04 | 22.38933333 | 1.089830338 |
| GO:0006915~apoptotic process | 20 | 8.26E-04 | 2.369241623 | 1.400770371 |
| GO:0002576~platelet degranulation | 8 | 8.57E-04 | 5.216932039 | 1.453013146 |
| GO:0051091~positive regulation of sequence-specific DNA binding transcription factor activity | 8 | 9.61E-04 | 5.117561905 | 1.626655399 |
| GO:0043085~positive regulation of catalytic activity | 7 | 0.001284174 | 5.804641975 | 2.16908021 |
| GO:0006468~protein phosphorylation | 17 | 0.001321192 | 2.504070175 | 2.230945146 |
| GO:0006364~rRNA processing | 11 | 0.001378797 | 3.452560748 | 2.32714044 |

| Top upregulated functions based on differential expression in S3 versus non-S3 tumors | Count | PValue | Fold Enrichment | FDR |
| --- | --- | --- | --- | --- |
| GO:0002576~platelet degranulation | 17 | 1.67E-11 | 9.758785724 | 2.85E-08 |
| GO:0055114~oxidation-reduction process | 37 | 2.48E-11 | 3.695422535 | 4.22E-08 |
| GO:0042730~fibrinolysis | 9 | 9.92E-10 | 25.34004024 | 1.69E-06 |
| GO:0006635~fatty acid beta-oxidation | 11 | 2.36E-09 | 14.78169014 | 4.02E-06 |
| GO:0055088~lipid homeostasis | 10 | 1.31E-08 | 15.16070784 | 2.24E-05 |
| GO:0033539~fatty acid beta-oxidation using acyl-CoA DH | 7 | 3.40E-07 | 22.99374022 | 5.80E-04 |
| GO:0006559~L-phenylalanine catabolic process | 6 | 5.58E-07 | 32.25096031 | 9.51E-04 |
| GO:0010951~negative regulation of endopeptidase activity | 13 | 9.51E-07 | 6.352461879 | 0.001619858 |
| GO:0006520~cellular amino acid metabolic process | 8 | 4.15E-06 | 11.82535211 | 0.00707326 |
| GO:0006805~xenobiotic metabolic process | 10 | 6.37E-06 | 7.580353918 | 0.010853044 |
| GO:0031639~plasminogen activation | 5 | 9.31E-06 | 32.84820031 | 0.015859123 |
| GO:0007597~blood coagulation, intrinsic pathway | 6 | 9.39E-06 | 19.70892019 | 0.016000634 |
| GO:0009636~response to toxic substance | 10 | 1.30E-05 | 6.956089478 | 0.022108705 |
| GO:0051918~negative regulation of fibrinolysis | 5 | 1.53E-05 | 29.56338028 | 0.026080105 |
| GO:0042493~response to drug | 18 | 1.67E-05 | 3.500926612 | 0.028428535 |
| GO:0006069~ethanol oxidation | 5 | 3.51E-05 | 24.63615023 | 0.059846669 |
| GO:0006695~cholesterol biosynthetic process | 7 | 3.81E-05 | 10.89177168 | 0.064915886 |
| GO:0006572~tyrosine catabolic process | 4 | 4.62E-05 | 47.30140845 | 0.078659675 |
| GO:0032496~response to lipopolysaccharide | 12 | 1.08E-04 | 4.326348334 | 0.183593096 |
| GO:0030168~platelet activation | 10 | 1.43E-04 | 5.14145744 | 0.243076378 |
| GO:0030855~epithelial cell differentiation | 8 | 1.74E-04 | 6.757344064 | 0.295244223 |
| GO:0006810~transport | 17 | 2.90E-04 | 2.888376234 | 0.4922843 |
| GO:0032869~cellular response to insulin stimulus | 8 | 3.15E-04 | 6.143040059 | 0.535257814 |
| GO:0010043~response to zinc ion | 6 | 3.22E-04 | 9.854460094 | 0.547922892 |
| GO:0045471~response to ethanol | 9 | 4.00E-04 | 5.068008048 | 0.67992556 |
| GO:0001523~retinoid metabolic process | 7 | 5.55E-04 | 6.785038097 | 0.94053746 |
| GO:0006081~cellular aldehyde metabolic process | 4 | 7.07E-04 | 21.5006402 | 1.197449915 |
| GO:0006094~gluconeogenesis | 6 | 8.32E-04 | 8.062740077 | 1.408594575 |
| GO:0030212~hyaluronan metabolic process | 4 | 9.31E-04 | 19.70892019 | 1.573934437 |
| GO:0006107~oxaloacetate metabolic process | 4 | 9.31E-04 | 19.70892019 | 1.573934437 |
| GO:0007596~blood coagulation | 11 | 0.001152302 | 3.53475199 | 1.944859039 |
| GO:2000145~regulation of cell motility | 5 | 0.001175468 | 10.5583501 | 1.983591255 |
| GO:0042311~vasodilation | 4 | 0.001502192 | 16.89336016 | 2.528322315 |
| GO:0030449~regulation of complement activation | 5 | 0.0015323 | 9.854460094 | 2.578376156 |
| GO:0001889~liver development | 7 | 0.00154232 | 5.593071945 | 2.595028866 |
| GO:0051919~positive regulation of fibrinolysis | 3 | 0.001660614 | 44.34507042 | 2.791424306 |
| GO:0019626~short-chain fatty acid catabolic process | 3 | 0.002736895 | 35.47605634 | 4.561254542 |
| GO:0019439~aromatic compound catabolic process | 3 | 0.002736895 | 35.47605634 | 4.561254542 |
| GO:0005975~carbohydrate metabolic process | 10 | 0.002829521 | 3.398089688 | 4.712142956 |
| GO:0055085~transmembrane transport | 12 | 0.002927661 | 2.90787347 | 4.871767182 |

Supplementary Table 2: At a FDR p-value cut-off of 0.25, significant enrichment was found for 7/50 gene sets from the mSigDB Hallmarks gene set collection.

| NAME | SIZE | ES | NES | NOM p-val | FDR q-val |
| --- | --- | --- | --- | --- | --- |
| HALLMARK_UNFOLDED_PROTEIN_RESPONSE | 113 | 0.4534 | 1.9199 | 0.0021 | 0.0689 |
| HALLMARK_MYC_TARGETS_V1 | 192 | 0.5180 | 1.8853 | 0.0233 | 0.0565 |
| HALLMARK_MITOTIC_SPINDLE | 196 | 0.4683 | 1.7926 | 0.0123 | 0.0879 |
| HALLMARK_MYC_TARGETS_V2 | 56 | 0.5989 | 1.7783 | 0.0149 | 0.0770 |
| HALLMARK_G2M_CHECKPOINT | 190 | 0.6629 | 1.7432 | 0.0062 | 0.0846 |
| HALLMARK_E2F_TARGETS | 189 | 0.6780 | 1.7103 | 0.0063 | 0.0945 |
| HALLMARK_DNA_REPAIR | 148 | 0.3280 | 1.6071 | 0.0842 | 0.1621 |

Supplementary Table 3: At a FDR p-value cut-off of 0.25, significant enrichment was found for 351/2675 gene sets from the C2:Chemical and Genetic Perturbation gene set collection.

| NAME | SIZE | ES | NES | NOM p-val | FDR q-val |
| --- | --- | --- | --- | --- | --- |
| MUELLER_PLURINET | 280 | 0.5245 | 1.9204 | 0.0042 | 0.0755 |
| PUIFFE_INVASION_INHIBITED_BY_ASCITES_UP | 79 | 0.4784 | 1.9164 | 0.0041 | 0.0758 |
| HONMA_DOCETAXEL_RESISTANCE | 33 | 0.4650 | 1.9248 | 0.0104 | 0.0760 |
| ZHAN_VARIABLE_EARLY_DIFFERENTIATION_GENES_DN | 28 | 0.5080 | 1.9396 | 0.0040 | 0.0792 |
| DANG_REGULATED_BY_MYC_UP | 67 | 0.5391 | 1.9079 | 0.0020 | 0.0793 |
| CAIRO_HEPATOBLASTOMA_UP | 192 | 0.5312 | 1.8972 | 0.0021 | 0.0808 |
| CHANG_CORE_SERUM_RESPONSE_UP | 196 | 0.4535 | 1.9260 | 0.0000 | 0.0808 |
| BASAKI_YBX1_TARGETS_UP | 270 | 0.6148 | 1.8934 | 0.0000 | 0.0809 |
| BENPORATH_ES_1 | 356 | 0.5570 | 1.9314 | 0.0000 | 0.0817 |
| SHAFFER_IRF4_TARGETS_IN_ACTIVATED_B_LYMPHOCYTE | 75 | 0.4544 | 1.8834 | 0.0000 | 0.0829 |
| TOYOTA_TARGETS_OF_MIR34B_AND_MIR34C | 409 | 0.5379 | 1.8771 | 0.0020 | 0.0830 |
| BLUM_RESPONSE_TO_SALIRASIB_DN | 325 | 0.5735 | 1.8977 | 0.0000 | 0.0841 |
| RHEIN_ALL_GLUCOCORTICOID_THERAPY_DN | 349 | 0.4245 | 1.8793 | 0.0083 | 0.0842 |
| PAL_PRMT5_TARGETS_UP | 192 | 0.4682 | 1.8856 | 0.0000 | 0.0844 |
| FUJII_YBX1_TARGETS_DN | 198 | 0.6675 | 1.8721 | 0.0000 | 0.0847 |
| WANG_RESPONSE_TO_GSK3_INHIBITOR_SB216763_DN | 337 | 0.6009 | 1.8630 | 0.0000 | 0.0851 |
| ZHANG_BREAST_CANCER_PROGENITORS_UP | 396 | 0.4905 | 1.8549 | 0.0082 | 0.0853 |
| MITSIADES_RESPONSE_TO_APLIDIN_DN | 239 | 0.5560 | 1.7912 | 0.0080 | 0.0854 |
| CHOW_RASSF1_TARGETS_UP | 26 | 0.5022 | 1.9409 | 0.0019 | 0.0857 |
| HORIUCHI_WTAP_TARGETS_DN | 283 | 0.5995 | 1.7961 | 0.0119 | 0.0859 |
| WINNEPENNINCKX_MELANOMA_METASTASIS_UP | 148 | 0.6627 | 1.8022 | 0.0060 | 0.0859 |
| PENG_RAPAMYCIN_RESPONSE_DN | 235 | 0.3598 | 1.8571 | 0.0186 | 0.0859 |
| RHODES_CANCER_META_SIGNATURE | 62 | 0.5138 | 1.7893 | 0.0060 | 0.0861 |
| SHEDDEN_LUNG_CANCER_POOR_SURVIVAL_A6 | 431 | 0.6459 | 1.7943 | 0.0020 | 0.0861 |
| KOBAYASHI_EGFR_SIGNALING_24HR_DN | 238 | 0.7202 | 1.7919 | 0.0000 | 0.0862 |
| BOYAULT_LIVER_CANCER_SUBCLASS_G3_UP | 183 | 0.5620 | 1.7489 | 0.0240 | 0.0862 |
| PUJANA_BRCA2_PCC_NETWORK | 404 | 0.5724 | 1.8673 | 0.0020 | 0.0866 |
| LIAO_METASTASIS | 489 | 0.5416 | 1.7475 | 0.0000 | 0.0867 |
| PEART_HDAC_PROLIFERATION_CLUSTER_UP | 54 | 0.5553 | 1.7965 | 0.0101 | 0.0868 |
| ZHOU_CELL_CYCLE_GENES_IN_IR_RESPONSE_6HR | 80 | 0.7511 | 1.7490 | 0.0020 | 0.0869 |
| CHIN_BREAST_CANCER_COPY_NUMBER_UP | 26 | 0.6082 | 1.7499 | 0.0000 | 0.0870 |
| SASAKI_ADULT_T_CELL_LEUKEMIA | 170 | 0.4617 | 1.7515 | 0.0063 | 0.0870 |
| ZHENG_GLIOBLASTOMA_PLASTICITY_UP | 242 | 0.5376 | 1.7462 | 0.0021 | 0.0871 |
| JAZAERI_BREAST_CANCER_BRCA1_VS_BRCA2_UP | 49 | 0.5002 | 1.8025 | 0.0021 | 0.0871 |
| HONRADO_BREAST_CANCER_BRCA1_VS_BRCA2 | 18 | 0.6741 | 1.7542 | 0.0021 | 0.0871 |
| PUJANA_BREAST_CANCER_LIT_INT_NETWORK | 99 | 0.5174 | 1.7922 | 0.0020 | 0.0873 |
| BROWNE_HCMV_INFECTION_14HR_UP | 145 | 0.4040 | 1.7976 | 0.0021 | 0.0873 |
| POMEROY_MEDULLOBLASTOMA_PROGNOSIS_DN | 42 | 0.5815 | 1.7531 | 0.0061 | 0.0874 |
| XU_HGF_TARGETS_INDUCED_BY_AKT1_48HR_DN | 25 | 0.7137 | 1.7503 | 0.0039 | 0.0874 |
| ZAMORA_NOS2_TARGETS_UP | 68 | 0.4959 | 1.8638 | 0.0079 | 0.0874 |
| JAIN_NFKB_SIGNALING | 73 | 0.4242 | 1.7868 | 0.0040 | 0.0876 |
| LI_WILMS_TUMOR_VS_FETAL_KIDNEY_1_DN | 157 | 0.6391 | 1.8058 | 0.0000 | 0.0876 |
| HOLLEMAN_VINCRISTINE_RESISTANCE_B_ALL_UP | 36 | 0.4991 | 1.8505 | 0.0038 | 0.0877 |
| WEST_ADRENOCORTICAL_TUMOR_UP | 275 | 0.4745 | 1.7441 | 0.0141 | 0.0877 |
| BENPORATH_PROLIFERATION | 137 | 0.6691 | 1.7545 | 0.0040 | 0.0878 |
| ZHOU_CELL_CYCLE_GENES_IN_IR_RESPONSE_24HR | 120 | 0.7321 | 1.7577 | 0.0000 | 0.0878 |
| OUELLET_CULTURED_OVARIAN_CANCER_INVASIVE_VS_LMP_UP | 66 | 0.4708 | 1.7515 | 0.0085 | 0.0879 |
| TARTE_PLASMA_CELL_VS_PLASMABLAST_DN | 299 | 0.4648 | 1.7584 | 0.0198 | 0.0880 |
| MANALO_HYPOXIA_DN | 268 | 0.5703 | 1.8030 | 0.0123 | 0.0881 |
| SMID_BREAST_CANCER_LUMINAL_A_DN | 17 | 0.8230 | 1.7444 | 0.0000 | 0.0882 |
| HOSHIDA_LIVER_CANCER_SUBCLASS_S2 | 113 | 0.4516 | 1.8041 | 0.0554 | 0.0884 |
| CHNG_MULTIPLE_MYELOMA_HYPERPLOID_UP | 52 | 0.4524 | 1.8065 | 0.0121 | 0.0884 |
| HIRSCH_CELLULAR_TRANSFORMATION_SIGNATURE_DN | 95 | 0.4336 | 1.7697 | 0.0000 | 0.0884 |
| PUJANA_XPRSS_INT_NETWORK | 160 | 0.6445 | 1.8471 | 0.0000 | 0.0884 |
| WHITFIELD_CELL_CYCLE_S | 146 | 0.5114 | 1.7424 | 0.0235 | 0.0884 |
| WHITFIELD_CELL_CYCLE_G2_M | 203 | 0.5169 | 1.7589 | 0.0079 | 0.0886 |
| GROSS_HYPOXIA_VIA_HIF1A_UP | 73 | 0.4073 | 1.7978 | 0.0041 | 0.0886 |
| BILANGES_RAPAMYCIN_SENSITIVE_VIA_TSC1_AND_TSC2 | 67 | 0.3682 | 1.8573 | 0.0132 | 0.0887 |
| IRITANI_MAD1_TARGETS_DN | 44 | 0.5143 | 1.7545 | 0.0284 | 0.0887 |
| LINDGREN_BLADDER_CANCER_CLUSTER_1_DN | 358 | 0.5021 | 1.7555 | 0.0040 | 0.0887 |
| WINTER_HYPOXIA_UP | 82 | 0.5533 | 1.7632 | 0.0061 | 0.0888 |
| RUIZ_TNC_TARGETS_DN | 136 | 0.6357 | 1.7610 | 0.0058 | 0.0889 |
| GARCIA_TARGETS_OF_FLI1_AND_DAX1_DN | 160 | 0.5270 | 1.7702 | 0.0041 | 0.0890 |
| GHO_ATF5_TARGETS_DN | 16 | 0.6878 | 1.8160 | 0.0040 | 0.0890 |
| SARRIO_EPITHELIAL_MESENCHYMAL_TRANSITION_UP | 163 | 0.6479 | 1.7839 | 0.0020 | 0.0891 |
| HOFFMANN_LARGE_TO_SMALL_PRE_BII_LYMPHOCYTE_UP | 158 | 0.6633 | 1.8089 | 0.0059 | 0.0891 |
| NUNODA_RESPONSE_TO_DASATINIB_IMATINIB_UP | 28 | 0.6368 | 1.7722 | 0.0081 | 0.0893 |
| LINDGREN_BLADDER_CANCER_CLUSTER_3_UP | 305 | 0.5296 | 1.7709 | 0.0060 | 0.0893 |
| JIANG_HYPOXIA_VIA_VHL | 31 | 0.4536 | 1.7592 | 0.0162 | 0.0894 |
| CHIARADONNA_NEOPLASTIC_TRANSFORMATION_KRAS_UP | 123 | 0.5503 | 1.8072 | 0.0000 | 0.0894 |
| PENG_GLUTAMINE_DEPRIVATION_DN | 319 | 0.3611 | 1.7732 | 0.0368 | 0.0894 |
| STEIN_ESRRA_TARGETS_DN | 97 | 0.3762 | 1.7743 | 0.0040 | 0.0896 |
| BIDUS_METASTASIS_UP | 200 | 0.5256 | 1.7403 | 0.0255 | 0.0896 |
| MALONEY_RESPONSE_TO_17AAG_DN | 74 | 0.4698 | 1.7611 | 0.0181 | 0.0897 |
| DUTERTRE_ESTRADIOL_RESPONSE_6HR_UP | 216 | 0.4511 | 1.7633 | 0.0041 | 0.0898 |
| GRADE_COLON_AND_RECTAL_CANCER_UP | 266 | 0.4043 | 1.8171 | 0.0119 | 0.0901 |
| TURASHVILI_BREAST_CARCINOMA_DUCTAL_VS_LOBULAR_UP | 19 | 0.6847 | 1.7804 | 0.0079 | 0.0901 |
| SENGUPTA_NASOPHARYNGEAL_CARCINOMA_UP | 273 | 0.5642 | 1.7792 | 0.0020 | 0.0903 |
| MISSIAGLIA_REGULATED_BY_METHYLATION_DN | 114 | 0.6308 | 1.7814 | 0.0081 | 0.0903 |
| NAKAYAMA_SOFT_TISSUE_TUMORS_PCA2_UP | 85 | 0.7796 | 1.7638 | 0.0000 | 0.0903 |
| DUTERTRE_ESTRADIOL_RESPONSE_24HR_UP | 309 | 0.6791 | 1.8093 | 0.0040 | 0.0905 |
| RICKMAN_TUMOR_DIFFERENTIATED_WELL_VS_POORLY_UP | 213 | 0.4273 | 1.7743 | 0.0127 | 0.0906 |
| VECCHI_GASTRIC_CANCER_EARLY_UP | 389 | 0.5719 | 1.7754 | 0.0040 | 0.0906 |
| GEORGES_CELL_CYCLE_MIR192_TARGETS | 58 | 0.6091 | 1.8430 | 0.0019 | 0.0907 |
| DACOSTA_UV_RESPONSE_VIA_ERCC3_UP | 296 | 0.3449 | 1.7380 | 0.0062 | 0.0908 |
| LE_EGR2_TARGETS_UP | 105 | 0.6621 | 1.7643 | 0.0021 | 0.0909 |
| GARGALOVIC_RESPONSE_TO_OXIDIZED_PHOSPHOLIPIDS_TURQUOISE_DN | 49 | 0.7182 | 1.7663 | 0.0020 | 0.0911 |
| FERREIRA_EWINGS_SARCOMA_UNSTABLE_VS_STABLE_UP | 157 | 0.6320 | 1.8101 | 0.0060 | 0.0914 |
| KAUFFMANN_DNA_REPLICATION_GENES | 136 | 0.4728 | 1.7757 | 0.0105 | 0.0915 |
| CUI_GLUCOSE_DEPRIVATION | 58 | 0.5457 | 1.7646 | 0.0062 | 0.0916 |
| FRASOR_RESPONSE_TO_SERM_OR_FULVESTRANT_DN | 49 | 0.7160 | 1.7312 | 0.0020 | 0.0917 |
| NADERI_BREAST_CANCER_PROGNOSIS_UP | 50 | 0.6698 | 1.7765 | 0.0021 | 0.0917 |
| ODONNELL_TFRC_TARGETS_DN | 127 | 0.7126 | 1.8174 | 0.0020 | 0.0919 |
| NIELSEN_SCHWANNOMA_UP | 17 | 0.7158 | 1.7318 | 0.0000 | 0.0920 |
| FERRANDO_T_ALL_WITH_MLL_ENL_FUSION_DN | 84 | 0.4643 | 1.7333 | 0.0124 | 0.0921 |
| WHITEFORD_PEDIATRIC_CANCER_MARKERS | 113 | 0.7371 | 1.7338 | 0.0040 | 0.0923 |
| WANG_CISPLATIN_RESPONSE_AND_XPC_UP | 193 | 0.5270 | 1.9505 | 0.0000 | 0.0923 |
| ABRAMSON_INTERACT_WITH_AIRE | 42 | 0.5978 | 1.7321 | 0.0179 | 0.0923 |
| GRAHAM_CML_DIVIDING_VS_NORMAL_QUIESCENT_UP | 175 | 0.6636 | 1.7288 | 0.0041 | 0.0925 |
| LY_AGING_OLD_DN | 55 | 0.7060 | 1.7296 | 0.0000 | 0.0925 |
| HU_GENOTOXIC_DAMAGE_24HR | 33 | 0.6037 | 1.7341 | 0.0040 | 0.0928 |
| SCHLOSSER_MYC_TARGETS_AND_SERUM_RESPONSE_DN | 46 | 0.5131 | 1.8104 | 0.0080 | 0.0929 |
| CHEMNITZ_RESPONSE_TO_PROSTAGLANDIN_E2_UP | 131 | 0.6168 | 1.7273 | 0.0118 | 0.0933 |
| SONG_TARGETS_OF_IE86_CMV_PROTEIN | 58 | 0.7250 | 1.7341 | 0.0061 | 0.0936 |
| WHITFIELD_CELL_CYCLE_G1_S | 126 | 0.5102 | 1.8175 | 0.0060 | 0.0937 |
| SCHLOSSER_MYC_TARGETS_AND_SERUM_RESPONSE_UP | 45 | 0.5328 | 1.9415 | 0.0021 | 0.0938 |
| PENG_LEUCINE_DEPRIVATION_DN | 175 | 0.3976 | 1.8194 | 0.0283 | 0.0939 |
| SHEPARD_CRUSH_AND_BURN_MUTANT_DN | 173 | 0.5276 | 1.7250 | 0.0041 | 0.0948 |
| SANSOM_APC_TARGETS_REQUIRE_MYC | 195 | 0.4406 | 1.8203 | 0.0021 | 0.0955 |
| WONG_EMBRYONIC_STEM_CELL_CORE | 323 | 0.5751 | 1.9568 | 0.0000 | 0.0960 |
| MORI_LARGE_PRE_BII_LYMPHOCYTE_UP | 83 | 0.6840 | 1.7202 | 0.0060 | 0.0963 |
| PEART_HDAC_PROLIFERATION_CLUSTER_DN | 74 | 0.5073 | 1.7079 | 0.0138 | 0.0965 |
| JIANG_TIP30_TARGETS_DN | 23 | 0.5565 | 1.7082 | 0.0199 | 0.0968 |
| KANG_FLUOROURACIL_RESISTANCE_DN | 16 | 0.6932 | 1.7219 | 0.0022 | 0.0970 |
| VERNELL_RETINOBLASTOMA_PATHWAY_UP | 68 | 0.6510 | 1.7204 | 0.0102 | 0.0970 |
| MORI_PRE_BI_LYMPHOCYTE_UP | 76 | 0.6388 | 1.7188 | 0.0102 | 0.0970 |
| BURTON_ADIPOGENESIS_PEAK_AT_16HR | 40 | 0.6743 | 1.8321 | 0.0000 | 0.0970 |
| PUJANA_BRCA_CENTERED_NETWORK | 114 | 0.6586 | 1.8210 | 0.0000 | 0.0971 |
| MARTINEZ_RESPONSE_TO_TRABECTEDIN_DN | 250 | 0.4353 | 1.9646 | 0.0078 | 0.0972 |
| GAVIN_FOXP3_TARGETS_CLUSTER_T7 | 95 | 0.3577 | 1.7084 | 0.0307 | 0.0973 |
| HEDENFALK_BREAST_CANCER_HEREDITARY_VS_SPORADIC | 49 | 0.4376 | 1.7179 | 0.0123 | 0.0974 |
| ZHANG_TLX_TARGETS_60HR_DN | 255 | 0.6364 | 1.7205 | 0.0103 | 0.0976 |
| SHAFFER_IRF4_TARGETS_IN_ACTIVATED_DENDRITIC_CELL | 61 | 0.4902 | 1.7088 | 0.0060 | 0.0976 |
| CAFFAREL_RESPONSE_TO_THC_DN | 27 | 0.5806 | 1.8288 | 0.0040 | 0.0978 |
| LEE_EARLY_T_LYMPHOCYTE_UP | 97 | 0.7580 | 1.7094 | 0.0078 | 0.0978 |
| DAZARD_RESPONSE_TO_UV_SCC_DN | 114 | 0.4303 | 1.8336 | 0.0040 | 0.0982 |
| RHODES_UNDIFFERENTIATED_CANCER | 67 | 0.6926 | 1.7139 | 0.0039 | 0.0985 |
| BILANGES_SERUM_SENSITIVE_VIA_TSC2 | 38 | 0.4948 | 1.7131 | 0.0119 | 0.0985 |
| BOYAULT_LIVER_CANCER_SUBCLASS_G123_UP | 44 | 0.6603 | 1.7116 | 0.0158 | 0.0985 |
| MORI_IMMATURE_B_LYMPHOCYTE_DN | 87 | 0.6851 | 1.7094 | 0.0100 | 0.0985 |
| HSIAO_HOUSEKEEPING_GENES | 384 | 0.2786 | 1.8217 | 0.0721 | 0.0987 |
| GOLUB_ALL_VS_AML_UP | 24 | 0.6352 | 1.7107 | 0.0127 | 0.0987 |
| SCHUHMACHER_MYC_TARGETS_UP | 78 | 0.4769 | 1.7151 | 0.0418 | 0.0988 |
| WU_APOPTOSIS_BY_CDKN1A_VIA_TP53 | 52 | 0.7836 | 1.7098 | 0.0020 | 0.0988 |
| SCHLOSSER_MYC_TARGETS_REPRESSED_BY_SERUM | 152 | 0.4388 | 1.7040 | 0.0508 | 0.0989 |
| CAFFAREL_RESPONSE_TO_THC_24HR_5_DN | 55 | 0.4600 | 1.7143 | 0.0167 | 0.0989 |
| TANG_SENESCENCE_TP53_TARGETS_DN | 55 | 0.7477 | 1.7118 | 0.0020 | 0.0991 |
| CAIRO_PML_TARGETS_BOUND_BY_MYC_UP | 23 | 0.6682 | 1.7010 | 0.0057 | 0.0991 |
| OXFORD_RALA_OR_RALB_TARGETS_UP | 47 | 0.7491 | 1.7003 | 0.0020 | 0.0991 |
| BURTON_ADIPOGENESIS_3 | 98 | 0.6935 | 1.7016 | 0.0099 | 0.0993 |
| WANG_TUMOR_INVASIVENESS_UP | 358 | 0.2909 | 1.8229 | 0.0020 | 0.0994 |
| DAIRKEE_CANCER_PRONE_RESPONSE_BPA_E2 | 116 | 0.4135 | 1.7153 | 0.0000 | 0.0994 |
| AFFAR_YY1_TARGETS_DN | 229 | 0.5102 | 1.6961 | 0.0041 | 0.0994 |
| CHANG_CYCLING_GENES | 137 | 0.7383 | 1.7027 | 0.0097 | 0.0994 |
| GOLDRATH_ANTIGEN_RESPONSE | 333 | 0.5208 | 1.7041 | 0.0080 | 0.0995 |
| PARK_HSC_AND_MULTIPOTENT_PROGENITORS | 46 | 0.3722 | 1.7020 | 0.0162 | 0.0995 |
| DANG_MYC_TARGETS_UP | 138 | 0.4008 | 1.6992 | 0.0398 | 0.0997 |
| KONG_E2F3_TARGETS | 96 | 0.7798 | 1.6964 | 0.0020 | 0.0998 |
| FOURNIER_ACINAR_DEVELOPMENT_LATE_2 | 261 | 0.5715 | 1.9757 | 0.0000 | 0.0998 |
| MARKEY_RB1_ACUTE_LOF_UP | 220 | 0.5832 | 1.8247 | 0.0060 | 0.0999 |
| JAEGER_METASTASIS_UP | 43 | 0.6471 | 1.6969 | 0.0020 | 0.0999 |
| LUI_THYROID_CANCER_CLUSTER_1 | 46 | 0.5193 | 1.6976 | 0.0105 | 0.1004 |
| CHIANG_LIVER_CANCER_SUBCLASS_PROLIFERATION_UP | 166 | 0.7951 | 1.6969 | 0.0000 | 0.1005 |
| AMUNDSON_GAMMA_RADIATION_RESPONSE | 39 | 0.8532 | 1.6933 | 0.0000 | 0.1013 |
| AIYAR_COBRA1_TARGETS_DN | 28 | 0.5532 | 1.6916 | 0.0135 | 0.1024 |
| YAMASHITA_LIVER_CANCER_WITH_EPCAM_UP | 52 | 0.5675 | 1.6889 | 0.0105 | 0.1044 |
| YAO_TEMPORAL_RESPONSE_TO_PROGESTERONE_CLUSTER_14 | 134 | 0.3383 | 1.6871 | 0.0231 | 0.1045 |
| VANDESLUIS_COMMD1_TARGETS_GROUP_4_UP | 19 | 0.6233 | 1.6878 | 0.0064 | 0.1049 |
| JOHANSSON_GLIOMAGENESIS_BY_PDGFB_UP | 53 | 0.5117 | 1.6872 | 0.0196 | 0.1050 |
| SPIELMAN_LYMPHOBLAST_EUROPEAN_VS_ASIAN_UP | 463 | 0.3047 | 1.6822 | 0.0168 | 0.1064 |
| WHITFIELD_CELL_CYCLE_G2 | 163 | 0.5172 | 1.6813 | 0.0161 | 0.1067 |
| BENPORATH_ES_2 | 34 | 0.6298 | 1.6824 | 0.0062 | 0.1068 |
| YAMAZAKI_TCEB3_TARGETS_DN | 204 | 0.4145 | 1.6825 | 0.0042 | 0.1073 |
| FOURNIER_ACINAR_DEVELOPMENT_LATE_DN | 21 | 0.7714 | 1.6830 | 0.0041 | 0.1074 |
| ZHAN_V2_LATE_DIFFERENTIATION_GENES | 43 | 0.5056 | 1.6833 | 0.0061 | 0.1077 |
| HU_GENOTOXIC_DAMAGE_4HR | 33 | 0.7341 | 1.6757 | 0.0020 | 0.1086 |
| MODY_HIPPOCAMPUS_PRENATAL | 40 | 0.4685 | 1.6785 | 0.0359 | 0.1087 |
| XU_HGF_SIGNALING_NOT_VIA_AKT1_48HR_DN | 17 | 0.7961 | 1.6757 | 0.0041 | 0.1092 |
| LY_AGING_PREMATURE_DN | 29 | 0.7097 | 1.6744 | 0.0020 | 0.1093 |
| ODONNELL_TARGETS_OF_MYC_AND_TFRC_DN | 43 | 0.7689 | 1.6766 | 0.0096 | 0.1096 |
| PUJANA_BREAST_CANCER_WITH_BRCA1_MUTATED_UP | 52 | 0.7031 | 1.6759 | 0.0080 | 0.1097 |
| AMUNDSON_GENOTOXIC_SIGNATURE | 101 | 0.4779 | 1.6767 | 0.0101 | 0.1102 |
| ISHIDA_E2F_TARGETS | 52 | 0.7689 | 1.6707 | 0.0041 | 0.1124 |
| GAVIN_FOXP3_TARGETS_CLUSTER_P6 | 87 | 0.6787 | 1.6707 | 0.0141 | 0.1130 |
| BILANGES_SERUM_RESPONSE_TRANSLATION | 35 | 0.4879 | 1.6682 | 0.0098 | 0.1146 |
| SAKAI_CHRONIC_HEPATITIS_VS_LIVER_CANCER_UP | 80 | 0.3719 | 1.6645 | 0.0301 | 0.1180 |
| CHIARETTI_T_ALL_RELAPSE_PROGNOSIS | 19 | 0.7111 | 1.6608 | 0.0082 | 0.1184 |
| MATTIOLI_MGUS_VS_PCL | 97 | 0.4344 | 1.6635 | 0.0248 | 0.1186 |
| YU_MYC_TARGETS_UP | 40 | 0.7506 | 1.6610 | 0.0058 | 0.1188 |
| NIELSEN_GIST_VS_SYNOVIAL_SARCOMA_UP | 19 | 0.8066 | 1.6612 | 0.0000 | 0.1192 |
| MENSSEN_MYC_TARGETS | 52 | 0.4275 | 1.6589 | 0.0444 | 0.1193 |
| WELCSH_BRCA1_TARGETS_DN | 135 | 0.4294 | 1.9762 | 0.0061 | 0.1194 |
| BENPORATH_ES_CORE_NINE_CORRELATED | 99 | 0.4938 | 1.6614 | 0.0123 | 0.1196 |
| BHATTACHARYA_EMBRYONIC_STEM_CELL | 86 | 0.5042 | 1.6591 | 0.0135 | 0.1197 |
| BORCZUK_MALIGNANT_MESOTHELIOMA_UP | 291 | 0.3383 | 1.6578 | 0.0624 | 0.1201 |
| MOLENAAR_TARGETS_OF_CCND1_AND_CDK4_DN | 52 | 0.7648 | 1.6614 | 0.0080 | 0.1203 |
| MARKS_HDAC_TARGETS_DN | 15 | 0.7801 | 1.6551 | 0.0020 | 0.1204 |
| IWANAGA_E2F1_TARGETS_INDUCED_BY_SERUM | 27 | 0.6386 | 1.6554 | 0.0277 | 0.1207 |
| GREENBAUM_E2A_TARGETS_UP | 33 | 0.7991 | 1.6555 | 0.0020 | 0.1211 |
| SHEPARD_BMYB_MORPHOLINO_DN | 187 | 0.4928 | 1.6524 | 0.0059 | 0.1212 |
| SHIPP_DLBCL_VS_FOLLICULAR_LYMPHOMA_UP | 44 | 0.6047 | 1.6538 | 0.0313 | 0.1214 |
| LOPEZ_MESOTHELIOMA_SURVIVAL_OVERALL_DN | 15 | 0.7690 | 1.6527 | 0.0078 | 0.1215 |
| SHEPARD_BMYB_TARGETS | 68 | 0.6257 | 1.6557 | 0.0181 | 0.1216 |
| EPPERT_LSC_R | 35 | 0.4674 | 1.6529 | 0.0123 | 0.1220 |
| COLDREN_GEFITINIB_RESISTANCE_UP | 72 | 0.4867 | 1.6514 | 0.0136 | 0.1220 |
| KIM_WT1_TARGETS_DN | 423 | 0.3836 | 1.6501 | 0.0267 | 0.1228 |
| CAFFAREL_RESPONSE_TO_THC_24HR_5_UP | 31 | 0.4712 | 1.6469 | 0.0388 | 0.1260 |
| GRAHAM_NORMAL_QUIESCENT_VS_NORMAL_DIVIDING_DN | 85 | 0.7216 | 1.6443 | 0.0139 | 0.1273 |
| GENTILE_UV_LOW_DOSE_UP | 27 | 0.5604 | 1.6438 | 0.0149 | 0.1274 |
| ZHANG_RESPONSE_TO_CANTHARIDIN_DN | 68 | 0.3938 | 1.6444 | 0.0698 | 0.1278 |
| MOREAUX_B_LYMPHOCYTE_MATURATION_BY_TACI_DN | 66 | 0.4956 | 1.6445 | 0.0585 | 0.1284 |
| HOLLEMAN_VINCRISTINE_RESISTANCE_ALL_UP | 24 | 0.4760 | 1.6417 | 0.0210 | 0.1287 |
| CROONQUIST_NRAS_VS_STROMAL_STIMULATION_DN | 96 | 0.6424 | 1.6419 | 0.0082 | 0.1291 |
| YU_BAP1_TARGETS | 26 | 0.6741 | 1.6403 | 0.0138 | 0.1297 |
| RIZ_ERYTHROID_DIFFERENTIATION | 77 | 0.5188 | 1.6358 | 0.0404 | 0.1321 |
| FLECHNER_PBL_KIDNEY_TRANSPLANT_REJECTED_VS_OK_UP | 62 | 0.3457 | 1.6359 | 0.0319 | 0.1325 |
| HESS_TARGETS_OF_HOXA9_AND_MEIS1_UP | 63 | 0.4548 | 1.6362 | 0.0227 | 0.1327 |
| SOTIRIOU_BREAST_CANCER_GRADE_1_VS_3_UP | 142 | 0.7591 | 1.6341 | 0.0175 | 0.1328 |
| ROSTY_CERVICAL_CANCER_PROLIFERATION_CLUSTER | 137 | 0.7981 | 1.6370 | 0.0059 | 0.1331 |
| HOLLEMAN_PREDNISOLONE_RESISTANCE_B_ALL_UP | 21 | 0.4714 | 1.6343 | 0.0466 | 0.1333 |
| HOFMANN_MYELODYSPLASTIC_SYNDROM_RISK_UP | 24 | 0.6235 | 1.6362 | 0.0211 | 0.1333 |
| YANG_BREAST_CANCER_ESR1_DN | 25 | 0.6846 | 1.6293 | 0.0042 | 0.1383 |
| PETROVA_PROX1_TARGETS_UP | 27 | 0.5919 | 1.6274 | 0.0282 | 0.1387 |
| REN_BOUND_BY_E2F | 59 | 0.6744 | 1.6283 | 0.0262 | 0.1387 |
| KARLSSON_TGFB1_TARGETS_UP | 116 | 0.3682 | 1.6270 | 0.0316 | 0.1387 |
| ZHANG_TLX_TARGETS_36HR_DN | 173 | 0.5876 | 1.6277 | 0.0570 | 0.1389 |
| SHAFFER_IRF4_MULTIPLE_MYELOMA_PROGRAM | 35 | 0.5373 | 1.6259 | 0.0381 | 0.1389 |
| BROWNE_HCMV_INFECTION_18HR_UP | 171 | 0.3913 | 1.6262 | 0.0023 | 0.1389 |
| CROONQUIST_NRAS_SIGNALING_DN | 71 | 0.7872 | 1.6249 | 0.0060 | 0.1397 |
| KAUFFMANN_MELANOMA_RELAPSE_UP | 57 | 0.6930 | 1.6203 | 0.0164 | 0.1434 |
| SHAFFER_IRF4_TARGETS_IN_PLASMA_CELL_VS_MATURE_B_LYMPHOCYTE | 66 | 0.5007 | 1.6197 | 0.0301 | 0.1435 |
| ZHANG_TLX_TARGETS_DN | 84 | 0.7137 | 1.6214 | 0.0317 | 0.1435 |
| JIANG_HYPOXIA_CANCER | 76 | 0.3440 | 1.6203 | 0.0122 | 0.1440 |
| SMID_BREAST_CANCER_RELAPSE_IN_LUNG_UP | 21 | 0.7297 | 1.6187 | 0.0041 | 0.1442 |
| LEE_LIVER_CANCER_SURVIVAL_DN | 166 | 0.5800 | 1.9784 | 0.0020 | 0.1472 |
| CROONQUIST_IL6_DEPRIVATION_DN | 95 | 0.7554 | 1.6155 | 0.0080 | 0.1479 |
| VANTVEER_BREAST_CANCER_BRCA1_UP | 33 | 0.5259 | 1.6143 | 0.0239 | 0.1488 |
| WILCOX_RESPONSE_TO_PROGESTERONE_UP | 139 | 0.4832 | 1.6123 | 0.0258 | 0.1507 |
| VANTVEER_BREAST_CANCER_METASTASIS_DN | 111 | 0.5981 | 1.6101 | 0.0400 | 0.1519 |
| LAU_APOPTOSIS_CDKN2A_UP | 55 | 0.3733 | 1.6102 | 0.0371 | 0.1524 |
| STEIN_ESRRA_TARGETS_RESPONSIVE_TO_ESTROGEN_DN | 41 | 0.6346 | 1.6047 | 0.0248 | 0.1528 |
| GARY_CD5_TARGETS_DN | 411 | 0.3516 | 1.6103 | 0.0588 | 0.1529 |
| LE_NEURONAL_DIFFERENTIATION_DN | 19 | 0.7747 | 1.6054 | 0.0118 | 0.1532 |
| YAO_TEMPORAL_RESPONSE_TO_PROGESTERONE_CLUSTER_12 | 76 | 0.3794 | 1.6049 | 0.0147 | 0.1532 |
| GROSS_HYPOXIA_VIA_ELK3_UP | 197 | 0.3637 | 1.6066 | 0.0240 | 0.1536 |
| ALONSO_METASTASIS_NEURAL_UP | 18 | 0.6659 | 1.6061 | 0.0223 | 0.1536 |
| SARTIPY_NORMAL_AT_INSULIN_RESISTANCE_UP | 32 | 0.6432 | 1.6054 | 0.0302 | 0.1538 |
| OZEN_MIR125B1_TARGETS | 24 | 0.5023 | 1.6067 | 0.0347 | 0.1541 |
| MORI_MATURE_B_LYMPHOCYTE_DN | 74 | 0.4756 | 1.6008 | 0.0335 | 0.1541 |
| FURUKAWA_DUSP6_TARGETS_PCI35_DN | 67 | 0.6446 | 1.6075 | 0.0339 | 0.1543 |
| ALONSO_METASTASIS_UP | 187 | 0.3261 | 1.6009 | 0.0349 | 0.1546 |
| BOHN_PRIMARY_IMMUNODEFICIENCY_SYNDROM_UP | 44 | 0.5108 | 1.6068 | 0.0356 | 0.1547 |
| BENPORATH_MYC_TARGETS_WITH_EBOX | 226 | 0.3216 | 1.6012 | 0.0083 | 0.1549 |
| HOLLEMAN_PREDNISOLONE_RESISTANCE_ALL_UP | 19 | 0.5152 | 1.6024 | 0.0362 | 0.1551 |
| BACOLOD_RESISTANCE_TO_ALKYLATING_AGENTS_DN | 54 | 0.4004 | 1.6013 | 0.0141 | 0.1554 |
| MARKS_ACETYLATED_NON_HISTONE_PROTEINS | 15 | 0.5515 | 1.6014 | 0.0493 | 0.1559 |
| WHITFIELD_CELL_CYCLE_LITERATURE | 42 | 0.8187 | 1.5978 | 0.0080 | 0.1575 |
| KAMMINGA_EZH2_TARGETS | 41 | 0.7147 | 1.5965 | 0.0060 | 0.1585 |
| GENTILE_UV_HIGH_DOSE_DN | 298 | 0.3640 | 1.5913 | 0.0554 | 0.1647 |
| DACOSTA_UV_RESPONSE_VIA_ERCC3_TTD_UP | 62 | 0.3780 | 1.5892 | 0.0189 | 0.1648 |
| BOYLAN_MULTIPLE_MYELOMA_C_CLUSTER_UP | 36 | 0.4561 | 1.5895 | 0.0102 | 0.1650 |
| CHAUHAN_RESPONSE_TO_METHOXYESTRADIOL_DN | 98 | 0.3581 | 1.5908 | 0.0690 | 0.1650 |
| SANSOM_APC_TARGETS | 193 | 0.4194 | 1.5902 | 0.0084 | 0.1653 |
| BILANGES_SERUM_AND_RAPAMYCIN_SENSITIVE_GENES | 68 | 0.3985 | 1.5897 | 0.0574 | 0.1654 |
| LIAO_HAVE_SOX4_BINDING_SITES | 40 | 0.6419 | 1.5870 | 0.0142 | 0.1656 |
| JUBAN_TARGETS_OF_SPI1_AND_FLI1_DN | 85 | 0.3799 | 1.5873 | 0.0217 | 0.1658 |
| BURTON_ADIPOGENESIS_PEAK_AT_24HR | 40 | 0.6771 | 1.5875 | 0.0394 | 0.1662 |
| SCIBETTA_KDM5B_TARGETS_DN | 77 | 0.4952 | 1.5845 | 0.0243 | 0.1667 |
| KANG_DOXORUBICIN_RESISTANCE_UP | 53 | 0.8208 | 1.5875 | 0.0060 | 0.1667 |
| KOKKINAKIS_METHIONINE_DEPRIVATION_48HR_UP | 123 | 0.4273 | 1.5847 | 0.0354 | 0.1670 |
| APPIERTO_RESPONSE_TO_FENRETINIDE_DN | 47 | 0.4260 | 1.5850 | 0.0318 | 0.1671 |
| AKL_HTLV1_INFECTION_UP | 25 | 0.4843 | 1.5854 | 0.0367 | 0.1671 |
| EPPERT_PROGENITOR | 131 | 0.4400 | 1.5830 | 0.0504 | 0.1680 |
| GENTILE_RESPONSE_CLUSTER_D3 | 59 | 0.5822 | 2.0187 | 0.0000 | 0.1702 |
| OUELLET_OVARIAN_CANCER_INVASIVE_VS_LMP_UP | 113 | 0.3433 | 1.5798 | 0.0878 | 0.1713 |
| DEBIASI_APOPTOSIS_BY_REOVIRUS_INFECTION_DN | 271 | 0.3355 | 1.5798 | 0.0146 | 0.1719 |
| VANDESLUIS_COMMD1_TARGETS_GROUP_3_UP | 82 | 0.4684 | 1.5764 | 0.0107 | 0.1738 |
| MOHANKUMAR_TLX1_TARGETS_UP | 394 | 0.3189 | 1.5765 | 0.0245 | 0.1743 |
| SCHLOSSER_MYC_AND_SERUM_RESPONSE_SYNERGY | 31 | 0.4319 | 1.5768 | 0.0785 | 0.1745 |
| ZHAN_MULTIPLE_MYELOMA_PR_UP | 44 | 0.8303 | 1.5772 | 0.0119 | 0.1745 |
| COLLER_MYC_TARGETS_UP | 24 | 0.5205 | 1.5722 | 0.0528 | 0.1778 |
| LI_WILMS_TUMOR_ANAPLASTIC_UP | 18 | 0.8134 | 1.5730 | 0.0121 | 0.1781 |
| KRASNOSELSKAYA_ILF3_TARGETS_DN | 44 | 0.5706 | 1.5724 | 0.0145 | 0.1781 |
| KARAKAS_TGFB1_SIGNALING | 18 | 0.7161 | 1.5711 | 0.0120 | 0.1788 |
| SU_TESTIS | 73 | 0.5279 | 1.5694 | 0.0454 | 0.1795 |
| RAHMAN_TP53_TARGETS_PHOSPHORYLATED | 21 | 0.4633 | 1.5696 | 0.0476 | 0.1797 |
| SMID_BREAST_CANCER_RELAPSE_IN_BRAIN_UP | 39 | 0.6548 | 1.5698 | 0.0063 | 0.1801 |
| HOFMANN_MYELODYSPLASTIC_SYNDROM_LOW_RISK_DN | 30 | 0.5652 | 1.5681 | 0.0261 | 0.1808 |
| LOPEZ_MBD_TARGETS_IMPRINTED_AND_X_LINKED | 17 | 0.6399 | 1.5670 | 0.0304 | 0.1817 |
| OUILLETTE_CLL_13Q14_DELETION_DN | 53 | 0.4851 | 1.5661 | 0.0105 | 0.1824 |
| FARMER_BREAST_CANCER_CLUSTER_2 | 32 | 0.8384 | 1.5651 | 0.0119 | 0.1825 |
| GARGALOVIC_RESPONSE_TO_OXIDIZED_PHOSPHOLIPIDS_GREEN_DN | 24 | 0.4693 | 1.5651 | 0.0245 | 0.1831 |
| THILLAINADESAN_ZNF217_TARGETS_UP | 40 | 0.4927 | 1.5615 | 0.0462 | 0.1853 |
| MMS_MOUSE_LYMPH_HIGH_4HRS_UP | 35 | 0.4877 | 1.5619 | 0.0656 | 0.1855 |
| CHIANG_LIVER_CANCER_SUBCLASS_UNANNOTATED_DN | 181 | 0.4079 | 1.5608 | 0.0982 | 0.1856 |
| WEST_ADRENOCORTICAL_TUMOR_MARKERS_UP | 20 | 0.6611 | 1.5621 | 0.0404 | 0.1857 |
| KYNG_RESPONSE_TO_H2O2_VIA_ERCC6 | 17 | 0.5286 | 1.5622 | 0.0396 | 0.1863 |
| TOOKER_GEMCITABINE_RESISTANCE_UP | 75 | 0.3094 | 1.5594 | 0.0214 | 0.1871 |
| LI_WILMS_TUMOR | 27 | 0.7337 | 1.5580 | 0.0101 | 0.1879 |
| CHICAS_RB1_TARGETS_GROWING | 233 | 0.4841 | 1.5584 | 0.0320 | 0.1880 |
| BILD_E2F3_ONCOGENIC_SIGNATURE | 216 | 0.4140 | 1.5574 | 0.0125 | 0.1881 |
| MORI_EMU_MYC_LYMPHOMA_BY_ONSET_TIME_UP | 94 | 0.5361 | 1.9826 | 0.0000 | 0.1893 |
| MARIADASON_RESPONSE_TO_CURCUMIN_SULINDAC_7 | 16 | 0.5588 | 1.5562 | 0.0438 | 0.1893 |
| MEINHOLD_OVARIAN_CANCER_LOW_GRADE_DN | 20 | 0.5932 | 1.5548 | 0.0318 | 0.1902 |
| LY_AGING_MIDDLE_DN | 16 | 0.9120 | 1.5551 | 0.0020 | 0.1904 |
| NIELSEN_SYNOVIAL_SARCOMA_UP | 18 | 0.7248 | 1.5535 | 0.0169 | 0.1915 |
| LEE_TARGETS_OF_PTCH1_AND_SUFU_UP | 49 | 0.5550 | 1.5523 | 0.0329 | 0.1928 |
| FIRESTEIN_CTNNB1_PATHWAY | 32 | 0.4506 | 1.5512 | 0.0542 | 0.1936 |
| SANSOM_APC_MYC_TARGETS | 203 | 0.3374 | 1.5503 | 0.0263 | 0.1936 |
| FOSTER_KDM1A_TARGETS_DN | 191 | 0.3117 | 1.5503 | 0.0061 | 0.1942 |
| ONO_FOXP3_TARGETS_DN | 41 | 0.6601 | 1.5427 | 0.0309 | 0.2050 |
| DAZARD_UV_RESPONSE_CLUSTER_G6 | 141 | 0.3938 | 1.5416 | 0.0487 | 0.2054 |
| BOYLAN_MULTIPLE_MYELOMA_C_UP | 43 | 0.4418 | 1.5420 | 0.0290 | 0.2055 |
| DEN_INTERACT_WITH_LCA5 | 26 | 0.4440 | 1.5388 | 0.0695 | 0.2087 |
| CERVERA_SDHB_TARGETS_1_DN | 35 | 0.6070 | 1.5388 | 0.0126 | 0.2094 |
| BHATI_G2M_ARREST_BY_2METHOXYESTRADIOL_UP | 115 | 0.4593 | 1.5358 | 0.0203 | 0.2095 |
| HEDENFALK_BREAST_CANCER_BRCA1_VS_BRCA2 | 156 | 0.3157 | 1.5361 | 0.0554 | 0.2096 |
| WANG_METHYLATED_IN_BREAST_CANCER | 35 | 0.5990 | 1.5363 | 0.0426 | 0.2099 |
| HAMAI_APOPTOSIS_VIA_TRAIL_DN | 179 | 0.4599 | 1.5363 | 0.0216 | 0.2106 |
| LIANG_HEMATOPOIESIS_STEM_CELL_NUMBER_QTL | 15 | 0.6162 | 1.5366 | 0.0472 | 0.2108 |
| DORSAM_HOXA9_TARGETS_UP | 33 | 0.4090 | 1.5369 | 0.0503 | 0.2110 |
| GRAHAM_CML_QUIESCENT_VS_NORMAL_QUIESCENT_UP | 83 | 0.5213 | 1.5333 | 0.0369 | 0.2121 |
| SMIRNOV_RESPONSE_TO_IR_6HR_DN | 107 | 0.6224 | 1.5334 | 0.0330 | 0.2125 |
| DAZARD_RESPONSE_TO_UV_NHEK_DN | 296 | 0.3258 | 1.5317 | 0.0609 | 0.2126 |
| CAFFAREL_RESPONSE_TO_THC_UP | 31 | 0.4058 | 1.5299 | 0.0942 | 0.2129 |
| REICHERT_MITOSIS_LIN9_TARGETS | 26 | 0.8002 | 1.5302 | 0.0154 | 0.2130 |
| ZHAN_EARLY_DIFFERENTIATION_GENES_DN | 42 | 0.5891 | 1.5310 | 0.0577 | 0.2131 |
| HOLLEMAN_VINCRISTINE_RESISTANCE_B_ALL_DN | 15 | 0.4863 | 1.5318 | 0.0686 | 0.2132 |
| COLLIS_PRKDC_SUBSTRATES | 19 | 0.4718 | 1.5303 | 0.0755 | 0.2136 |
| SLEBOS_HEAD_AND_NECK_CANCER_WITH_HPV_UP | 75 | 0.4913 | 1.5319 | 0.0585 | 0.2138 |
| DAZARD_RESPONSE_TO_UV_SCC_UP | 113 | 0.3775 | 1.5264 | 0.0756 | 0.2176 |
| KYNG_RESPONSE_TO_H2O2_VIA_ERCC6_DN | 46 | 0.3608 | 1.5243 | 0.0351 | 0.2201 |
| WILLIAMS_ESR1_TARGETS_UP | 26 | 0.5735 | 1.5236 | 0.0148 | 0.2206 |
| JIANG_VHL_TARGETS | 122 | 0.3127 | 1.5225 | 0.0507 | 0.2216 |
| SWEET_KRAS_TARGETS_DN | 63 | 0.4696 | 1.5202 | 0.0125 | 0.2227 |
| JISON_SICKLE_CELL_DISEASE_DN | 163 | 0.3170 | 1.5212 | 0.0404 | 0.2231 |
| KIM_TIAL1_TARGETS | 32 | 0.4455 | 1.5202 | 0.0494 | 0.2234 |
| PYEON_CANCER_HEAD_AND_NECK_VS_CERVICAL_UP | 167 | 0.4508 | 1.5203 | 0.0842 | 0.2240 |
| SIMBULAN_UV_RESPONSE_IMMORTALIZED_DN | 30 | 0.5322 | 1.5176 | 0.0374 | 0.2264 |
| BOYAULT_LIVER_CANCER_SUBCLASS_G23_UP | 50 | 0.6151 | 1.5165 | 0.0786 | 0.2277 |
| MUNSHI_MULTIPLE_MYELOMA_UP | 78 | 0.3773 | 1.5161 | 0.0688 | 0.2277 |
| HUMMEL_BURKITTS_LYMPHOMA_UP | 38 | 0.5223 | 1.5134 | 0.0181 | 0.2309 |
| PARK_HSC_MARKERS | 41 | 0.4916 | 1.5137 | 0.0701 | 0.2310 |
| HOLLEMAN_ASPARAGINASE_RESISTANCE_B_ALL_UP | 26 | 0.4859 | 1.5088 | 0.0936 | 0.2379 |
| SMITH_TERT_TARGETS_UP | 140 | 0.3559 | 1.5081 | 0.0717 | 0.2380 |
| BONCI_TARGETS_OF_MIR15A_AND_MIR16_1 | 88 | 0.4411 | 1.5082 | 0.0273 | 0.2383 |
| SANA_RESPONSE_TO_IFNG_DN | 82 | 0.4173 | 1.5069 | 0.0507 | 0.2385 |
| STEIN_ESR1_TARGETS | 85 | 0.4641 | 1.5071 | 0.0325 | 0.2389 |
| GUENTHER_GROWTH_SPHERICAL_VS_ADHERENT_UP | 20 | 0.6266 | 1.5052 | 0.0104 | 0.2392 |
| ALCALAY_AML_BY_NPM1_LOCALIZATION_DN | 179 | 0.4506 | 1.5046 | 0.0327 | 0.2394 |
| GENTILE_UV_RESPONSE_CLUSTER_D2 | 40 | 0.4843 | 1.5055 | 0.0870 | 0.2394 |
| AMIT_EGF_RESPONSE_480_MCF10A | 42 | 0.5094 | 1.5056 | 0.0467 | 0.2400 |
| CUI_TCF21_TARGETS_2_UP | 397 | 0.4452 | 1.5028 | 0.0220 | 0.2405 |
| SENESE_HDAC3_TARGETS_DN | 470 | 0.3396 | 1.5031 | 0.0150 | 0.2407 |
| CONCANNON_APOPTOSIS_BY_EPOXOMICIN_DN | 161 | 0.4608 | 1.5032 | 0.0385 | 0.2412 |
| EGUCHI_CELL_CYCLE_RB1_TARGETS | 23 | 0.8157 | 1.5005 | 0.0178 | 0.2436 |
| MATTHEWS_SKIN_CARCINOGENESIS_VIA_JUN | 15 | 0.6366 | 1.5001 | 0.0439 | 0.2436 |
| GENTILE_UV_RESPONSE_CLUSTER_D4 | 52 | 0.3669 | 1.4980 | 0.0626 | 0.2467 |
| KUUSELO_PANCREATIC_CANCER_19Q13_AMPLIFICATION | 28 | 0.5080 | 1.4962 | 0.0441 | 0.2491 |
| SHIN_B_CELL_LYMPHOMA_CLUSTER_8 | 36 | 0.5674 | 1.4952 | 0.0441 | 0.2493 |

Supplementary Figure 1: Heat map of the top ranked genes resulting from the mSigDB Hallmarks collection. Columns correspond to samples (grey = high AFP, yellow = low AFP) and rows correspond to genes.


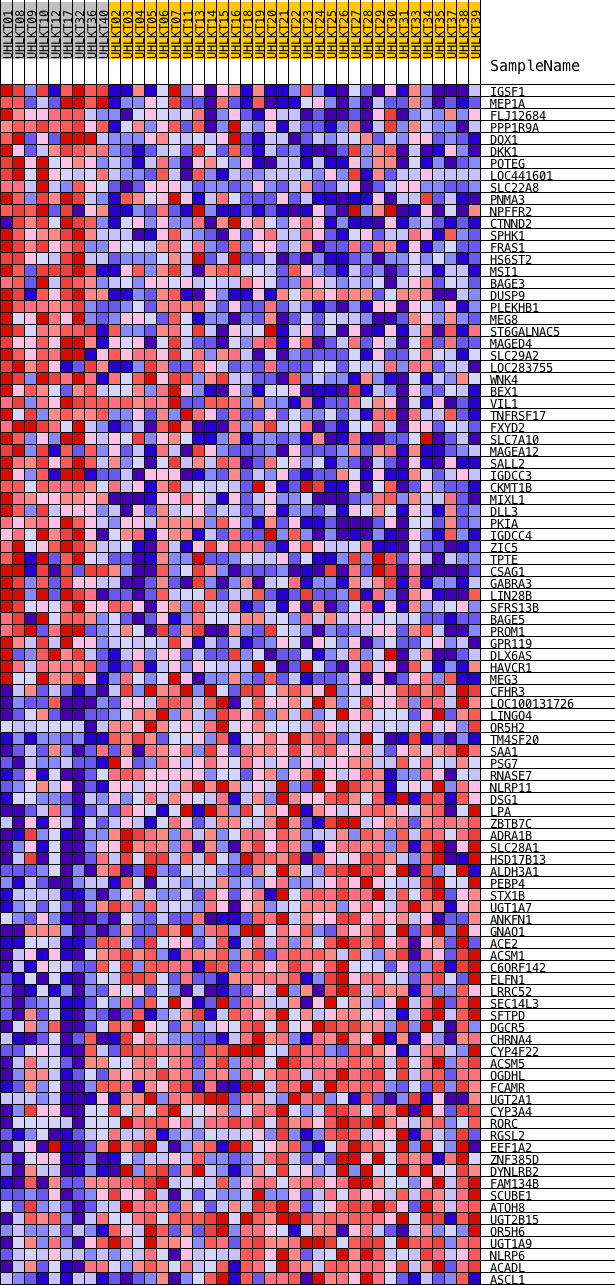


Supplementary Figure 2: Heat map of the top ranked genes resulting from C2:Chemical and Genetic Perturbations collection. Columns correspond to samples (grey = high AFP, yellow = low AFP) and rows correspond to genes.


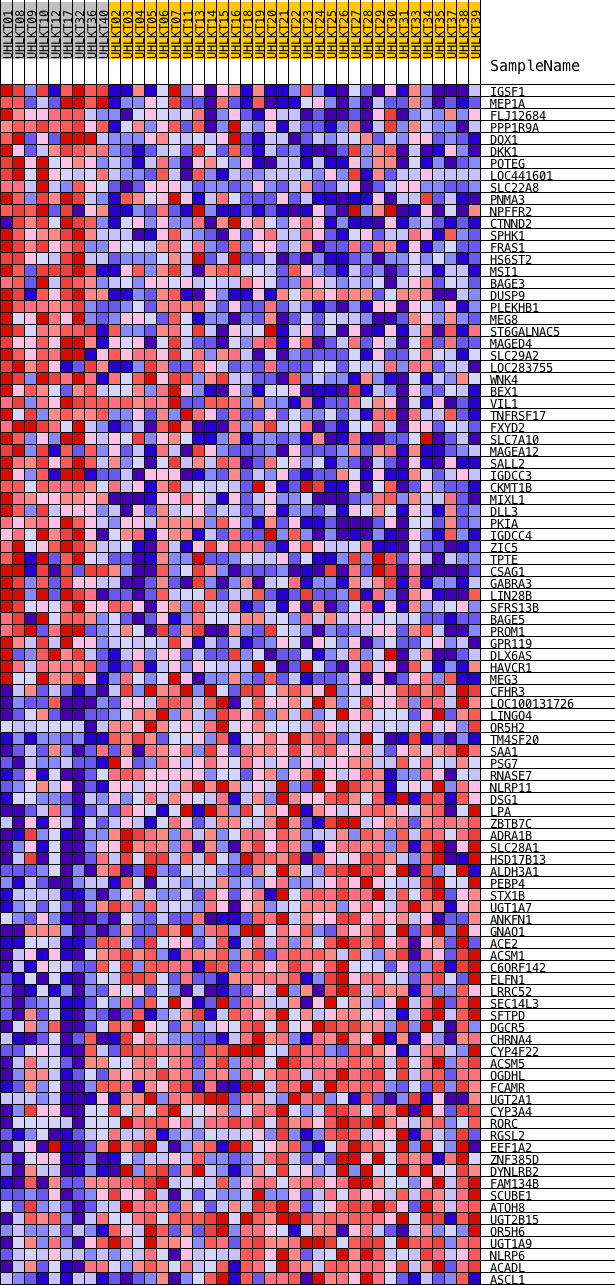

Supplement: Supplemental Results [file NIHMS934591-supplement-Supplemental_Results.docx]
